# Quantum dots reveal heterogeneous membrane diffusivity and dynamic surface density polarization of dopamine transporter

**DOI:** 10.1371/journal.pone.0225339

**Published:** 2019-11-21

**Authors:** Oleg Kovtun, Ian D. Tomlinson, Riley S. Ferguson, Sandra J. Rosenthal

**Affiliations:** 1 Department of Chemistry, Vanderbilt University, Nashville, Tennessee, United States of America; 2 Vanderbilt Institute of Nanoscale Science and Engineering, Vanderbilt University, Nashville, Tennessee, United States of America; 3 Department of Pharmacology, Vanderbilt University, Nashville, Tennessee, United States of America; 4 Department of Chemical and Biomolecular Engineering, Vanderbilt University, Nashville, Tennessee, United States of America; 5 Department of Physics and Astronomy, Vanderbilt University, Nashville, Tennessee, United States of America; 6 Vanderbilt Institute of Chemical Biology, Vanderbilt University, Nashville, Tennessee, United States of America; Beijing Forestry University, CHINA

## Abstract

The presynaptic dopamine transporter mediates rapid reuptake of synaptic dopamine. Although cell surface DAT trafficking recently emerged as an important component of DAT regulation, it has not been systematically investigated. Here, we apply our single quantum dot (Qdot) tracking approach to monitor DAT plasma membrane dynamics in several heterologous expression cell hosts with nanometer localization accuracy. We demonstrate that Qdot-tagged DAT proteins exhibited highly heterogeneous membrane diffusivity dependent on the local membrane topography. We also show that Qdot-tagged DATs were localized away from the flat membrane regions and were dynamically retained in the membrane protrusions and cell edges for the duration of imaging. Single quantum dot tracking of wildtype DAT and its conformation-defective coding variants (R60A and W63A) revealed a significantly accelerated rate of dysfunctional DAT membrane diffusion. We believe our results warrant an in-depth investigation as to whether compromised membrane dynamics is a common feature of brain disorder-derived DAT mutants.

## Introduction

The presynaptic dopamine transporter (DAT) encoded by the *SLC6A3* gene is responsible for Na^+^-dependent reuptake of extracellular dopamine released from the nerve terminals and therefore is a key mediator of dopaminergic neurotransmission [1–4]. Genetic variation in the *SLC6A3* gene has been linked to various neuropsychiatric disorders, including bipolar disorder, schizophrenia, attention deficit/hyperactivity disorder (ADHD), and Parkinson’s disease [5–9]. DAT is also a principal target for illicit drugs of abuse (cocaine, amphetamine) and ADHD medications (Adderall, Ritalin) [3,4]. As there are no extracellular enzymes that catabolize dopamine upon its release at the presynaptic active zone, the duration of dopamine transient signal is primarily controlled by the rapid dopamine diffusion away from the active zone to the extrasynaptic areas and subsequent reuptake of dopamine through the perisynaptic DATs [1,2,10]. Considering the slow transport cycle of DAT (1–5 dopamine molecules/s) [10] relative to the rapid timescale of synaptic dopamine signals, proper DAT membrane localization is a key prerequisite for efficient dopamine clearance.

The molecular mechanisms underlying DAT membrane organization and surface trafficking have been a subject of intense scrutiny over the past decade [11–15]. Ensemble and single-molecule fluorescence microscopy techniques have been pivotal to our understanding of the contribution of DAT surface trafficking to the regulation of dopamine homeostasis; it is now clear that DAT is a dynamic entity in the plasma membrane sensitive to local microenvironment manipulation and capable of engaging various direct binding partners [12–15]. Specifically, Adkins *et al*. [11] employed fluorescence recovery after photobleaching (FRAP) and fluorescence correlation spectroscopy (FCS) to demonstrate that yellow fluorescent protein (YFP)-fused DAT accelerated laterally after acute cholesterol depletion with methyl-β-cyclodextrin and actin cytoskeleton disruption in HEK293 and N2A cells. The increase in the DAT surface diffusion rate was accompanied by a significant reduction in the rate of [^3^H]dopamine uptake, pointing to an important link between DAT membrane microdomain localization and the transport component of the DAT function. Cremona *et al*. [13] discovered that flotillin, an intracellular scaffolding protein, was required to maintain a large fraction of DAT in membrane microdomains and facilitate protein kinase C (PKC)-triggered DAT internalization. Eriksen and colleagues surprisingly observed via FRAP that a large pool of fluorescent cocaine analog-occupied DATs exhibited non-directional, unrestricted Brownian diffusion in the midbrain neuron extensions and varicosities [14]. In contrast, Rahbek-Clemmensen *et al*. [15] implemented a stochastic optical reconstruction microscopy (STORM) approach to reveal that 50–70% of immunolabeled DAT proteins exist as irregular nanoclusters with a median size of 70 nm both in transfected CAD cells and the network of neuronal extensions. Additionally, DAT coding variation appears to have a profound impact on DAT surface trafficking. Specifically, R60A and W63A substitutions, which target a conserved intracellular interaction network responsible for the inward-to-outward DAT transitions and result in a reduced DAT affinity for its substrates, were demonstrated to increase the membrane pool of mobile DAT and accelerate its constitutive endocytosis [16–18]. A rare ADHD-derived R615C coding variation in the distal DAT C-terminus was reported to compromise DAT membrane stability and alter its recycling rate, possibly through mislocalization to GM1 ganglioside-rich microdomains [19]. Although the available data appear to point to the functional importance of DAT surface trafficking and DAT sequestration into specialized membrane microdomains, heterogeneity of DAT membrane organization at the individual protein level has not been systematically investigated.

We have previously developed a quantum dot-based imaging approach that allowed us to directly observe the membrane diffusion dynamics of surface DAT proteins [20,21]. Quantum dots are nanometer-sized semiconductor crystals that are characterized by excellent brightness, superior photostability compared to conventional fluorophores, broad absorption spectra, and narrow, size-tunable, Gaussian emission spectra [22]. Together, these signature photophysical properties of Qdots readily enable single particle tracking (SPT) of individual proteins with millisecond temporal and nanometer spatial resolution [23–25]. The Qdot surface can be readily derivatized with antibodies [26,27], peptides [28], or small-molecule ligands [29–31] to enable specific recognition of cell surface proteins. Here, we build upon our previous work and report a systematic study of the lateral motion of DAT in various live cell hosts. A central finding of this investigation is that DAT surface diffusion is highly heterogeneous and strongly dependent on the local membrane landscape. We also demonstrate that DAT is dynamically retained away from the flat membrane regions in all expression systems in a conformation-dependent manner, independently of the targeting strategy. We propose that the spatiotemporal polarization of DAT surface density occurs via a diffusion-based, curvature-dependent mechanism and may have important implications for psychiatric disorders associated with DAT coding variation.

## Materials and methods

### Materials

DMEM FluoroBrite Live cell imaging medium, dialyzed fetal bovine serum, Lipofectamine 3000, nocodazole, latrunculin B, m-3M3FBS, o-3M3FBS, and Qdot605Sav (emission max at 605 nm) were purchased from ThermoFisher Scientific. According to the manufacturer, the Qdot streptavidin conjugate is made from a nanometer-scale crystal of a semiconductor material (CdSe), which is coated with an additional semiconductor shell (ZnS) to improve the optical properties of the material. This core-shell material is further coated with a polymer shell that allows the materials to be conjugated to biological molecules and to retain their optical properties. This polymer shell is directly coupled to streptavidin through the PEG linker. The Qdot streptavidin conjugate is the size of a large macromolecule or protein (~15–20 nm). Poly-D-lysine hydrobromide (mol wt 70,000–150,000) and anti-HA-Biotin (High Affinity (3F10)) from rat IgG1 were purchased from Sigma-Aldrich. 35-mm uncoated No. 1.5 coverslip-bottomed dishes were purchased from MatTek. pcDNA3-EGFP plasmid DNA was a gift from Doug Golenbock (Addgene plasmid # 13031). pcDNA3.1-GFP-DRD2 plasmid DNA was a gift from Dr. Jean-Michel Arrang (Addgene plasmid # 24099). pcDNA3.1-GFP-DRD2 plasmid DNA was a gift from Dr. Jean-Michel Arrang (Addgene plasmid # 24099). tagRFP-C1-RFP-HA-DAT, pEYFP-C1-YFP-HA-DAT R60A, and pEYFP-C1-YFP-HA-DAT W63A plasmid DNA were a gift from Dr. Alexander Sorkin (Addgene plasmid # 90265, 90245, and 90246 respectively). pcDNA3.1-D2DR-3xHA was acquired from the cDNA Resource Center. The IDT772 ligand was synthesized as previously described [32].

### Cell culture

HEK-293, HEK-293T, and N2A cells were grown in a complete medium (DMEM with 2 mM glutamine, 10% FBS, 1% pen/strep) in a 37°C incubator with 5% CO_2_. CAD cells were grown in a complete medium (DMEM/Ham’s F12 1:1 with 2 mM glutamine, 8% FBS, 1% pen/strep) in a 37 °C incubator with 5% CO_2_. PC12 cells were grown in a complete medium (DMEM with 2 mM glutamine, 5% FBS, 5% horse serum, 1% pen/strep) in a 37 °C incubator with 5% CO_2_. Cells were seeded in poly-D-lysine coated (1 hr at 37 °C) MatTek dishes at an appropriate density to obtain a subconfluent monolayer and grown for 24 h in the appropriate complete growth medium. Then the cells were transiently transfected with 500 ng of the appropriate DNA per MatTek dish using Lipofectamine 3000 according to the manufacturer’s instructions.

### Qdot605Sav labeling

Qdot labeling was implemented via a two-step protocol. After the cells were allowed 24 hours to achieve transporter or receptor expression, labeling was carried out by first incubating the cells with IDT444 at 100 nM or anti-HA-biotin at 0.2 μg/mL for 10 minutes at 37 °C. Following two washes with warm DMEM FluoroBrite, cells were then incubated with a 0.02–0.10 nM Qdot605Sav diluted in warm DMEM FluoroBrite supplemented with 2% dFBS (labeling buffer) for 5 minutes at room temperature, washed three times with warm DMEM FluoroBrite, and used immediately for time-lapse image series acquisition.

### High-Speed spinning disk confocal microscopy

Time-lapse image series were obtained on an inverted Nikon-Ti Eclipse microscope system equipped with the Yokogawa CSU-X1 spinning disk confocal scanner unit, a heated stage, a 60x oil-immersion Plan Apo 1.4 NA objective, and the Andor DU-897 electron-multiplying charged-coupled device (EMCCD) camera. Qdots were excited using a 405-nm solid state diode laser (23 mW), and the Qdot emission was collected through the 605/70 emission filter. GFP/YFP molecules were excited using the 488-nm line (65 mW), and the GFP/YFP emission was collected using the 525/36 emission filter. RFP molecules were excited using the 561-nm line (86 mW), and the RFP emission was collected using the 605/70 emission filter. Single Qdot tracking was performed at a scan rate of 10–33 Hz for 1 minute. SPT data were obtained within 20 minutes of the final wash step after Qdot labeling.

### SIM microscopy

SIM imaging was performed in single-plane 3D SIM mode on an inverted Nikon SIM microscope equipped Andor DU-897 EMCCD camera, a SR Apo TIRF 100x 1.49 NA oil-immersion objective, and 488 nm (74 mW) and 561 nm (78 mW) solid-state diode lasers used to excited GFP/YFP and RFP fluorophores respectively. All cells were washed three times with room-temperature DMEM Fluorobrite and imaged in warm DMEM Fluorobrite at room temperature. The following treatments were performed on HEK-293T cells–o-3M3FBS (10 μM for 10 min), m-3M3FBS (10 μM for 10 min), latrunculin B (2.5 μM for 10 min), methyl-β-cyclodextrin (5 mM for 30 min), and nocodazole (10 μM for 1 hr).

### Data analysis

Image analysis and trajectory construction were performed using MATLAB according to the algorithms developed by Jaqaman *et al*. [33]. The localization accuracy of the central position of the Qdot in our imaging approach was estimated to be was estimated to be 25±10 nm based on 2411 Qdot trajectories immobilized onto a coverslip. Intermittency of Qdot fluorescence was used to verify that single fluorophores were analyzed, and extracted trajectories were at least 50 frames in length to increase the robustness of statistical analysis. Trajectories were considered continuous if a blinking Qdot was rediscovered within a 1 μm distance during the 10-frame time window. For each trajectory, mean square displacement (MSD), *r*^2^(*t*), was computed as follows:

$$\langle r^{2}(n\delta t)\rangle =\frac{1}{N-n}\sum_{j=0}^{N-n-1}\left\{{\left[x(j\delta t+n\delta t)-x(j\delta t)\right]}^{2}+{\left[y(j\delta t+n\delta t)-y(j\delta t)\right]}^{2}\right\}$$ (1)

where *δt* is the temporal resolution of the acquisition device, (*x*(*jδt*), *x*(*jδt*)) is the particle coordinate at t = *jδt*, and N is the number of total frames recorded for an individual particle. Prior to MSD calculations, individual trajectories were reindexed with continuous time vectors to close the gaps caused by blinking and simplify MSD analysis. Anomalous diffusion parameter (α) was estimated by fitting each individual MSD over time curve to 〈*r*^2^(*nδt*)〉 = 4*D*_*α*_*t*^∝^ using nonlinear least-squares fit [23]. The diffusion coefficient *D*_*MLE*_ was determined through the use of a previously published Maximum Likelihood Estimation (MLE) theoretical framework to maximize performance in accurately calculating D [34]. Trajectories with *D*_*MLE*_ < 5x10^-4^ μm^2^/s (equivalent to the 95^th^ percentile value of *D*_*MLE*_ derived from the analysis of 2411 trajectories of Qdots immobilized on glass coverslip) were considered immobilized and were omitted from further analysis. For each trajectory, the aspect ratio was calculated as the ratio of length to width of a minimum bounding box encompassing a trajectory.

### Relative deviation analysis to classify trajectory motion type

Independently of the motion mode, microscopic diffusion coefficient *D*_*2-4*_ was determined by fitting the first 2–4 points of the MSD versus time curves with the equation:

$${\langle r^{2}(t)\rangle }_{2-4}=4D_{2-4}t+offset$$ (2)

Four modes of motion are considered in order to describe the motional behavior of integral membrane proteins in the plasma membrane. These motional modes can be characterized on the basis of the plot of MSD versus time intervals:

1. Stationary (immobilized) mode, in which a protein displays very little motion. As this type of motion was indistinguishable from Qdots immobilized on glass substrate and comprised less than 5% of total trajectory number for each condition, Qdots trajectories classified as immobilized were omitted from further analysis as discussed in the section above.
2. Simple diffusion mode, in which a protein undergoes simple Brownian motion and its MSD-Δt plot is linear with a slope of 4D.
3. Directed diffusion mode, in which a protein moves in a direction with a constant drift velocity with superimposed random diffusion with a diffusion coefficient D. In this case, the MSD-Δt plot is parabolic with a differential coefficient of 4D at time 0 (initial slope).
4. Restricted diffusion mode, in which a protein undergoes Brownian motion within a limited area and cannot escape the area during the observation period (*0 ≤ x ≤ L*_*x*_, *0 ≤ y ≤ L*_*y*_). This mode of motion is equivalent to free Brownian diffusion within an infinitely high square well potential. The slope of the MSD-Δt curve at time 0 is again 4D, and the MSD-Δt curve asymptotically approaches *L*_*x*_^*2*^*/6* and *L*_*y*_^*2*^*/6* in the x and y directions, respectively.

The MSD-At plot shows positive and negative deviations from a straight line with a slope of 4D (in the case of two-dimensional diffusion) for directed diffusion and restricted diffusion, respectively. Larger deviations indicate larger probabilities of non-Brownian diffusion. A parameter for the relative deviation, RD(N, n), is defined as:

$$RD(N,n)=\frac{MSD(N,n)}{4D_{2-4}n\delta t}$$ (3)

where *MSD(N*, *n)* represents MSD determined at a time interval *n*δ*t* from a sequence of *N* video frames. *4D*_*2-4*_*nδt* is the expected average value of MSD for particles undergoing simple diffusion with a diffusion coefficient of *D*_*2-4*_ in two-dimensional space. In the case of simple diffusion, the average *RD(N*, *n)* should be 1 [35]. Brownian trajectories with a diffusion coefficient of 0.1 μm^2^/s were generated by random walk simulations using experimentally relevant trajectory lengths (100, 200, 300, 400, 500, and 600 steps) to establish the effective cutoff values of RD at 25 frames (S1 Fig). RD values within the 2.5^th^-97.5^th^ percentile range were taken to represent statistical variations in Brownian motion, and those outside of the range taken as restricted diffusion. S1 Fig maps the 2.5^th^ and 97.5^th^ percentiles of RD(N,25) as a function of N. A linear least-squares fit to the 2.5th percentile points defined the lower boundary for free diffusion, with trajectories having RD(N,25) below this line classified as restricted (S1 Fig). Since DAT at the plasma membrane should not be actively directed by any cellular processes, directed diffusion was ignored, so trajectories with RD(N,25) above the 4^th^-order polynomial fit of the 97.5th percentile points were classified as free [36].

### HMM analysis

In a single dimension, a particle trajectory consists of a sequence of particle positions x_t_ separated by a time interval Δt. For a particle undergoing a random walk, the particle instantaneous displacements Δx_t_ = x_t+1_−x_t_ along this dimension follow a normal distribution with a standard deviation that depends on the diffusion coefficient D_σ_ according to σ^2^ = 2D_σ_Δt and a mean that depends on the velocity v according to μ = vΔt. For a 2D particle trajectory with particle positions r_t_ = {x_t_, y_t_}, the instantaneous displacements between two consecutive frames become Δr_t_ = {Δx_t_, Δy_t_} = {x_t+1_ − x_t_, y_t+1_ − y_t_}. Hidden Markov Modeling analysis of a two-dimensional array of instantaneous displacements {Δx, Δy} for a single particle trajectory was implemented in Matlab using HMM-Bayes algorithm package available for download as shareware at http://hmm-bayes.org/ [37].

### Statistical analysis

Statistical pairwise comparison of differences between the distributions of individual diffusion coefficients and trajectory areas of Qdot-tagged DAT variants was carried out using Mann-Whitney U-test in Matlab R2018a (Mathworks, Inc., Natick, MA), Kolmogorov-Smirnov test in Matlab R2018a (Mathworks, Inc., Natick, MA), and one-way ANOVA with Dunnett’s post-hoc test in Sigma Plot 11 (Systat Software, Inc., San Jose, CA). Significance was set at α = 0.01. Diffusion data are represented as median with 25–75% interquartile interval.

## Results

### Single particle tracking of DAT lateral motion

**Fig 1:**
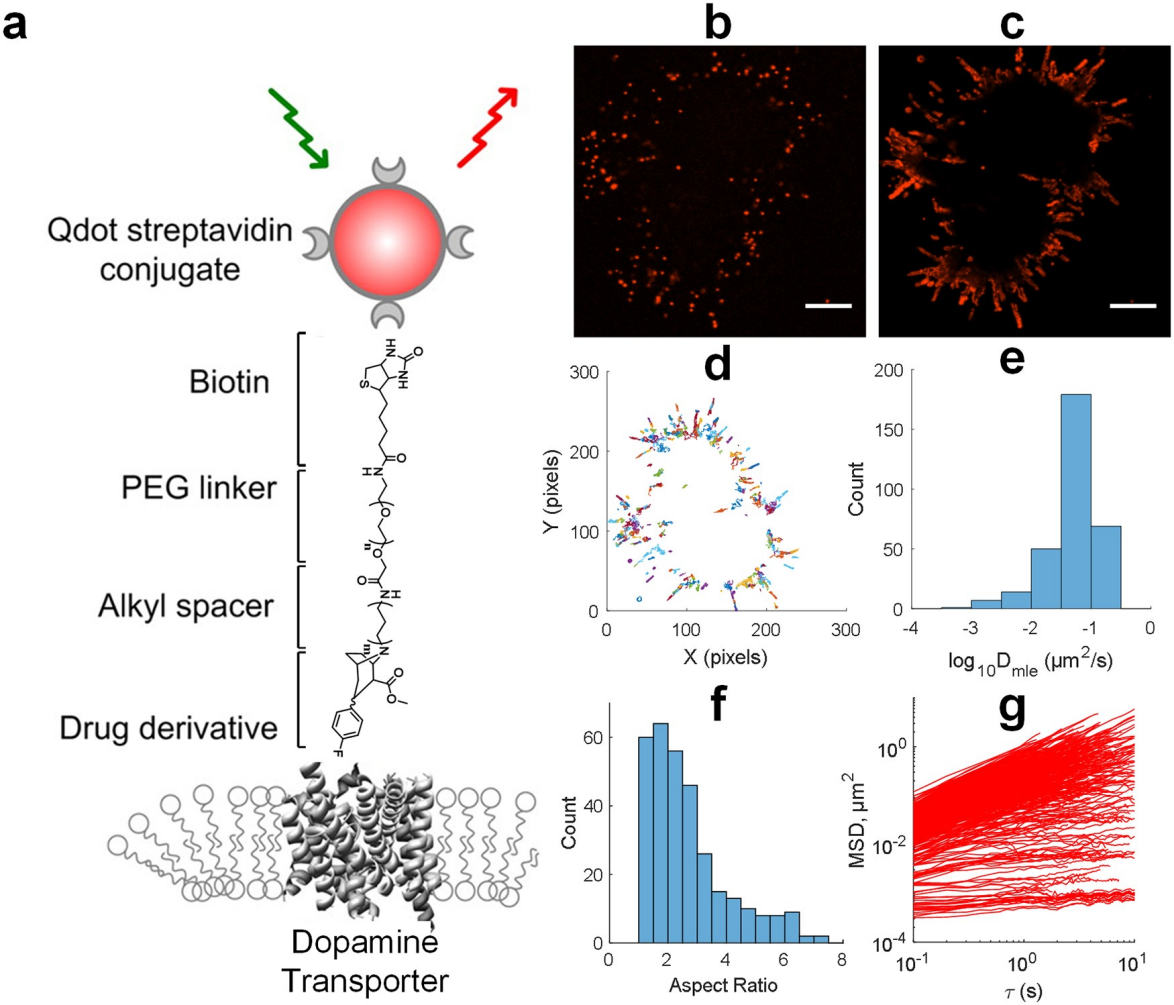
Single Qdot tracking reveals DAT heterogeneous diffusion dynamics. (a) The schematic illustrates the DAT-Qdot targeting strategy that relies on the use of biotinylated IDT444 ligand [20]. (b) A single frame of DAT-Qdot distribution at the basolateral membrane of HEK-293 cells; the image is representative of more than 5 independent experiments. Scale bar: 10 μm. (c) A maximum intensity projection of a 60-s time-lapse sequence of DAT-Qdots shown in b acquired at 10 Hz. Scale bar: 10 μm. (d) Reconstructed trajectories depicting DAT-Qdot motion in c. (e) A histogram of a diffusion coefficients *D*_*MLE*_ corresponding to trajectories in d. (f) A histogram of aspect ratios corresponding to individual trajectories in d. (g) A plot of individual MSD over time curves corresponding to individual trajectories in d and illustrating DAT-Qdot heterogeneous diffusion dynamics at the surface of a single cell examined.

We took advantage of our antagonist-conjugated Qdot labeling approach to obtain the trajectories of DAT proteins at the cell surface of heterologous expression systems (Fig 1). Our targeting strategy [20,21,23] features an organic ligand IDT444 composed of (i) a high-affinity cocaine analog that enables specific binding to the cell surface DAT molecules, (ii) an 11-carbon alkyl spacer to enable access to the binding site, (iii) a PEG chain to impart the ligand with aqueous solubility, and (iv) the biotin end which is captured by commercially available streptavidin-conjugated Qdots with the emission maximum at 605 nm (Qdot605Sav) (Fig 1a; refer to [20] for IDT444 synthesis details). Our ligand-based approach enables labeling and tracking of genetically unmodified transporters in live cells, as it has been notoriously difficult to develop an efficient antibody against a native DAT extracellular epitope [14]. Our strategy also eliminates the need for either a fluorescent protein usually fused to the DAT N-terminus or an epitope tag (e.g., hemagglutinin (HA), FLAG, or ligase acceptor peptide (LAP)) typically incorporated into the second extracellular loop (EL2) [16–18,38,39]. Since DAT has been shown to undergo modest (≤10%) constitutive internalization in a one-hour period both in transfected cells and *in vivo* [40], we are thus able to reliably observe the surface-limited trafficking events and probe DAT membrane dynamics at a spatial resolution of ~20 nm.

In the initial set of experiments, DAT-expressing HEK293 cells were sequentially labeled with a saturating dose of biotinylated IDT444 (100 nM) and a dilute solution of Qdot605Sav (0.1 nM). Trajectories were acquired for ~1 min at 10–33 Hz on the Nikon Ti-E inverted microscope platform equipped with the Yokogawa CSU-X1 spinning disk confocal scanner unit, a 60x oil-immersion Plan Apo 1.4 NA objective, and the Andor DU-897 EMCCD camera (S1 File). Fig 1b shows a representative frame of ~300 DAT trajectories over a time interval of 60 s at the basolateral surface (at the cell-coverslip interface) of transiently transfected HEK293 cells adhered to the poly-D-lysine-coated MatTek dish. Fig 1c shows a maximum intensity projection (600 frames, 1 min) of the time-lapse image series in Fig 1b. A maximum intensity projection was produced by selecting the highest intensity value for each pixel in a 2D image plane over the entire time-lapse sequence; it is a convenient way to visualize relative Qdot motion using unprocessed image series. Note that Qdots are localized to the periphery of the cells, and DAT lateral diffusion is primarily restricted to the cell edge. To obtain high-resolution DAT tracks, we relied on the tracking algorithm adapted from Jaqaman *et al*. [33] to retain trajectories of single, blinking Qdots that are at least 50 frames in duration. Shorter trajectories were discarded during analysis to increase the statistical robustness of data. Fig 1d shows trajectories derived from the representative time-lapse image series shown in Fig 1b and 1c. For each DAT-Qdot trajectory, we computed individual mean-square displacement (MSD) over time curves, an aspect ratio defined as the ratio of length to width of a minimum bounding box encompassing a trajectory, and individual diffusion coefficients (*D*_*MLE*_) according to a maximum likelihood estimation (MLE) algorithm [34] (Fig 1e, 1f and 1g). Maximum likelihood estimator takes into account the particle diffusion coefficient D, the static localization error σ, and the uncertainty due to motion blur R (refer to [34] for a detailed description of the likelihood estimator). The cutoff for immobilized Qdots was set to 5 x 10^−4^ μm^2^/s, equivalent to the 95% percentile of *D*_*MLE*_ distribution for 2411 immobilized Qdots that were spin-coated onto a coverslip. Analysis of the dynamic parameters of motion for these representative, biochemically de-facto identical Qdot-tagged DATs revealed a large amount of heterogeneity as evidenced by the large variation in the trajectory aspect ratio and MSD curves spanning several orders of magnitude.

### Resolving heterogeneity at the individual trajectory level

**Fig 2:**
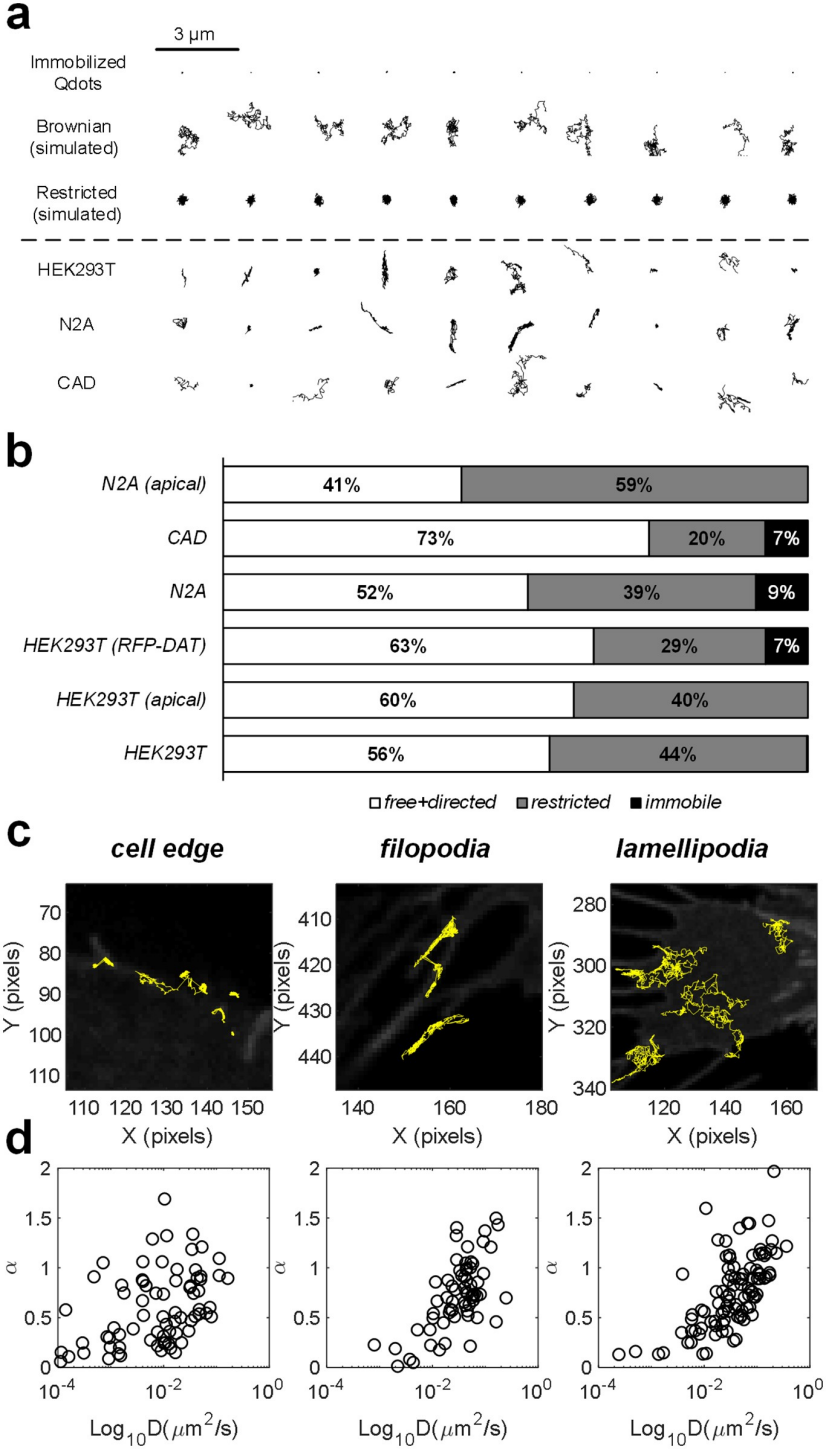
Analysis of DAT-Qdot diffusive heterogeneity at the single trajectory level. (a) A set of 10 random trajectories is shown for both simulated Brownian and restricted type of motion as well as experimentally acquired spin-coated (immobilized) Qdots and DAT-Qdots diffusing laterally at the surface of various cell hosts. (b) Classification of the type of motion of experimentally acquired DAT-Qdot trajectories using relative deviation analysis (HEK-293T/RFF-DAT: 2861 trajectories; HEK-293T/WT-DAT: 1236 trajectories; HEK-293T/WT-DAT on apical surface: 597 trajectories; N2A/WT-DAT: 1343 trajectories; N2A/WT-DAT on apical surface: 922 trajectories; CAD/WT-DAT: 1440 trajectories. Data were generated from three independent experiments. Two MatTek dishes with three 512x512 fields of view acquired per MatTek dish constituted an independent experiment. Two MatTek dishes were plated on separate days (per passage) for each cell line. (c) Representative trajectories of DAT-Qdots diffusig laterally in different membrane regions/topography. 1 pixel = 0.22 μm. (d) α parameter versus DMLE scatter plots for DAT-Qdots diffusing in three distinct membrane regions (N_edge_ = 79 tracks; N_filopodia_ = 66 tracks; N_lamellipodia_ = 100 tracks. Source data are provided in S1 Dataset.

Closer inspection of single DAT-Qdot trajectories in transiently transfected HEK-293T, N2A, and CAD cells revealed dynamic heterogeneity at the trajectory level. Fig 2a shows ten representative DAT-Qdot trajectories for each cell type randomly selected from the total pool. To gain a more quantitative description of the dynamic heterogeneity, we generated 1000 trajectories undergoing Brownian diffusion by random walk simulations with the rate of diffusion equivalent to that of DAT-Qdots in HEK-293T cells and 1000 trajectories confined to a parabolic potential with a trap size of 200 nm diffusing at the rate of DAT-Qdots in HEK-293T (Fig 2a). Next, we classified experimentally acquired DAT-Qdot trajectories by the relative deviation method [35,36]. The relative deviation parameter is defined as the ratio of the experimental MSD to the line extrapolated from the initial slope of MSD. For particles diffusing at a rate faster than the immobile *D*_*MLE*_ threshold, we analyzed relative deviation parameter at 25 frames to classify experimental trajectories as either free or restricted. Since plasma membrane DAT should not be actively transported by any cellular processes, directed diffusion was ignored [36]. Fig 2b summarizes the classification of the diffusion mode of the experimentally acquired DAT-Qdot trajectories. Notably, most trajectories were determined to constitute the mobile pool of plasma membrane DATs, exhibiting free Brownian motion. The restricted fraction varied from 20% to ~60% and appeared to be dependent on the expression host. Interestingly, lower number of DAT-Qdots in the freely diffusing pool at both the basolateral and apical surfaces of N2A cells is consistent with the previous finding by Adkins *et al*. [11] that observed markedly reduced YFP-DAT mobility in N2A versus HEK-293 cells via FRAP.

To shed light on the underlying mechanisms of DAT diffusion heterogeneity, we assessed the dependence of DAT-Qdot motion on DAT localization to distinct membrane features. In agreement with prior electron microscopy and immunocytochemistry studies indicating DAT enrichment in the network of neuronal extensions and varicosities relative to the weaker somatic signal [15,41], we observed relatively few DAT-Qdots diffusing in the flat membrane regions formed at the glass-cell interface of the three cell types examined. Most DAT-Qdots were localized to three distinct plasma membrane features–(i) thin, finger-like protrusions (filopodia), (ii) thin, flat membrane sheets (lamellipodia), and (iii) the membrane edge between flat membrane regions and features described in (i) and (ii). Fig 2c displays representative trajectories of DAT-Qdots from these three distinct membrane features. Whereas DAT-Qdots diffusing along the cell edge were characterized by the high degree of apparent confinement, most of the complexes localized to the finger-like membrane protrusions exhibited unobstructed bidirectional movement with frequent switching and those localized to the lamellipodia appeared to diffuse freely over the entire lamellipodia area without evident barriers. Next, we manually sorted at least 60 DAT-Qdot trajectories from different cell regions of HEK-293T cells and extracted two complementary parameters. First, we estimated the lateral mobility at short timescales by computing *D*_*MLE*_ for each individual trajectory. Second, we estimated the lateral mobility at longer timescales by computing the anomalous diffusion exponent α, a measure of by fitting the entire individual time-averaged MSD curves with a general model of diffusion MSD ≈ t^α^ [23]. The distribution of the parameters *D*_*MLE*_ and α extracted from single DAT-Qdot trajectories was represented as a two-dimensional scatter plot for each distinct membrane region (Fig 2d). As expected, the cell edge DAT-Qdots were diffusing at a significantly slower rate with a strongly subdiffusive α parameter than DAT-Qdots localized to the membrane lamellipodia or protrusions (Table 1). Thus, it appears that the heterogeneity in DAT-Qdot lateral mobility and its deviation from Brownian diffusion are strongly dependent on DAT residence in various membrane features.

**Table 1:** Cell region dependence of diffusion parameters.

Parameter	Cell Region	Median	25–75% IQR	N	Mann-Whitney U-test	K-S test
*D*_*MLE*_	edge	0.010 μm^2^/s	0.001–0.032 μm^2^/s	79	-----	-----
	filopodia	0.042 μm^2^/s	0.020–0.056 μm^2^/s	66	p = 4x10^-8^	p = 2x10^-7^
	lamellipodia	0.035 μm^2^/s	0.014–0.079 μm^2^/s	100	p = 4x10^-10^	p = 8 x10^-9^
*α*	edge	0.51	0.25–0.87	79	-----	-----
	filopodia	0.73	0.53–0.97	66	p = 0.0069	p = 0.0026
	lamellipodia	0.75	0.45–1.01	100	p = 2 x10^-4^	p = 0.0027

### Validation of polarized DAT-Qdot distribution

**Fig 3:**
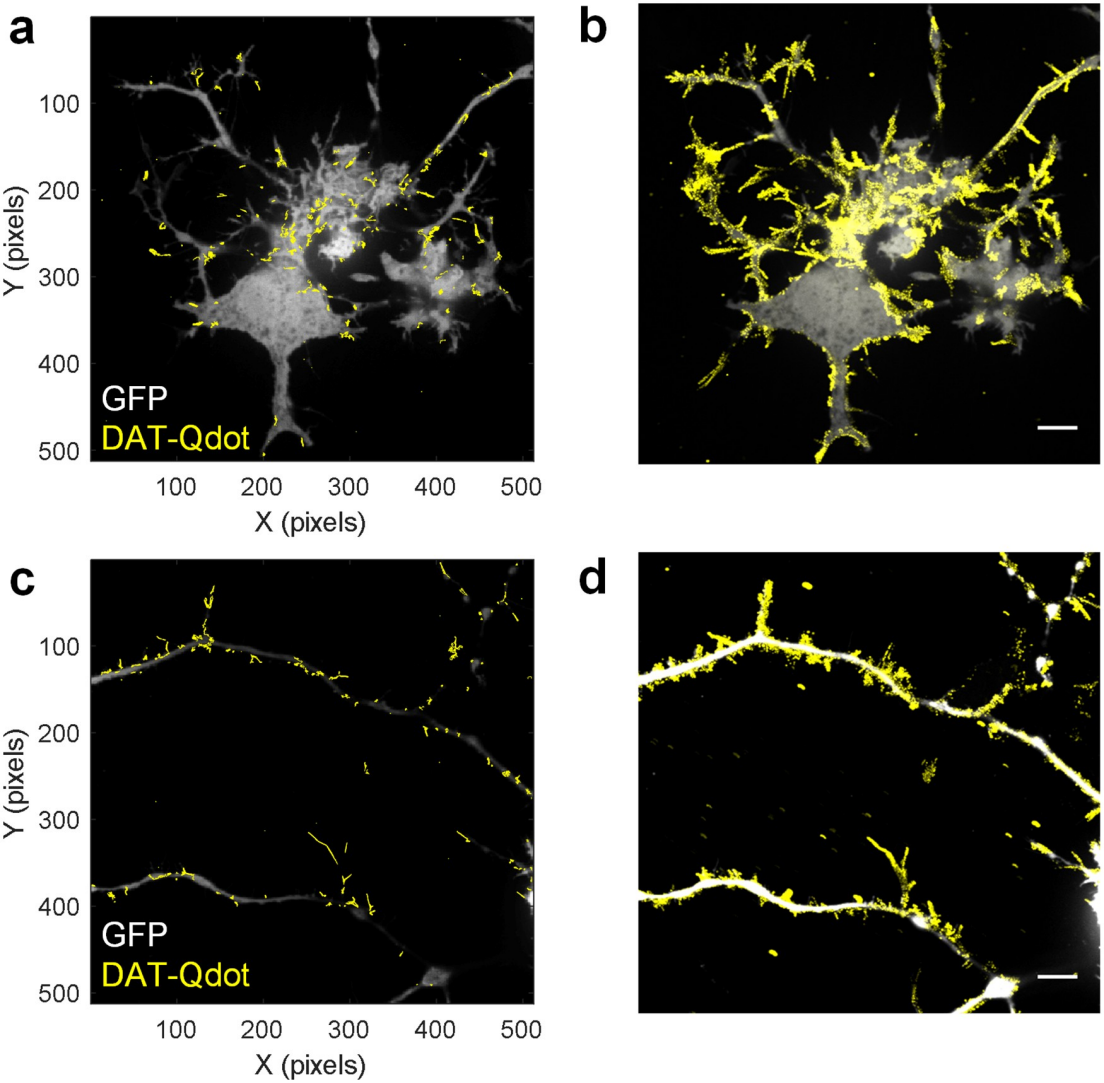
Avoidance of flat membrane regions by diffusing DAT-Qdots in N2A cells. Panels in a and c show DAT-Qdot motion over 60 s acquired at 10 Hz in GFP-expressing N2A cells. Maximum intensity projections in b and d show DAT-Qdot localizations acquired over 30 min at 0.1 Hz in GFP-expressing N2A cells. Scale bar:10 μm. Images are representative of 3 independent experiments. Two MatTek dishes plated per passage with three to six 512x512 fields of view acquired per MatTek dish constituted an independent experiment.

Highly polarized surface distribution and delivery of the plasma membrane molecules is a ubiquitous biological phenomenon [42–46]. The asymmetric and coordinated targeting and sorting of the cell surface molecules overcomes the randomizing effects of the Brownian motion that is predicted by the fluid mosaic model [47]. Molecular segregation at the cell surface is typically achieved through rigid diffusion barriers, dynamic confinement within the specialized membrane nanodomains, or preferential recruitment of molecules to the curved membrane regions [42–46]. Although DAT is strategically localized to the distal axonal processes of dopaminergic neurons to facilitate dopamine reuptake, it remains unclear whether DAT delivery to these areas is surface diffusion-based or relies on vesicular transport. Moreover, multiple lines of evidence indicate that the low density of DAT in the somatic region is independent of the cell host or the heterologous expression level [15,17,18,41]. Indeed, when we coexpressed DAT with the green fluorescent protein (GFP; not fused to DAT) to clearly outline membrane features in N2A cells, DAT-Qdot trajectories over a 60-s time period were mostly localized to the cell edges and thin membrane protrusions extending out of the flat membrane region (Fig 3a, S2 File) or the long neuritic outgrowths (Fig 3c, S3 File). Longer recordings of DAT-Qdot behavior at 0.1 Hz rate over a 30-min time period revealed additional distinctions. Maximum intensity projections of 30-min time-lapse image sequences showed that a large percentage of membrane protrusions were explored by DAT-Qdots, whereas the boundary between the flat membrane zone and the protrusions/processes in the same focal plane appeared to limit DAT-Qdot penetration into the flat membrane zone (Fig 3b and 3d, S4 and S5 Files). Isolated immobilized Qdots adsorbed to the coverslip surface served as a reference marker and assured the observation of DAT-Qdots at the plasma membrane/coverslip interface.

**Fig 4:**
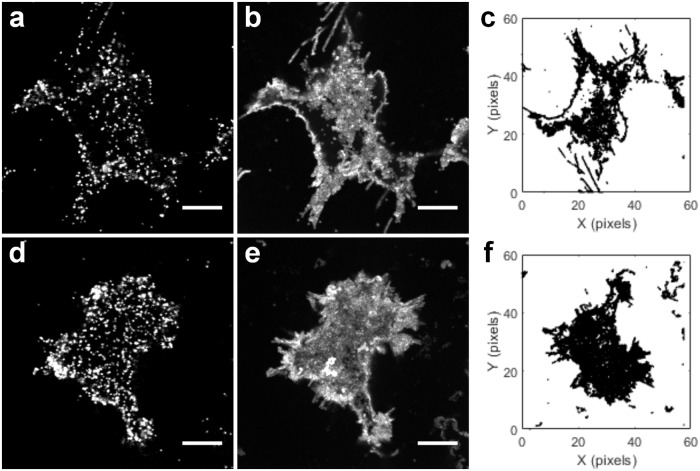
Uniformity of D2DR-Qdot lateral diffusion at the cell-coverslip interface independently of the labeling approach. (a) A single frame showing HA-tagged D2DR labeled with anti-HA-biotin and Qdot605Sav. Scale bar: 10 μm. (b) A maximum intensity projection showing D2DR-Qdot motion over 60 s acquired at 10 Hz. Scale bar: 10 μm. (c) Reconstructed trajectories depicting D2DR-Qdot motion in b. Scale: 1 pixel = 0.22 μm. (d-f) D2DR-HA labeling and tracking with biotinylated IDT772 and Qdot605Sav in HEK-293T cells. Images are representative of three independent experiments. Two MatTek dishes plated per passage with two to three 512x512 fields of view acquired per MatTek dish constituted an independent experiment.

**Fig 5:**
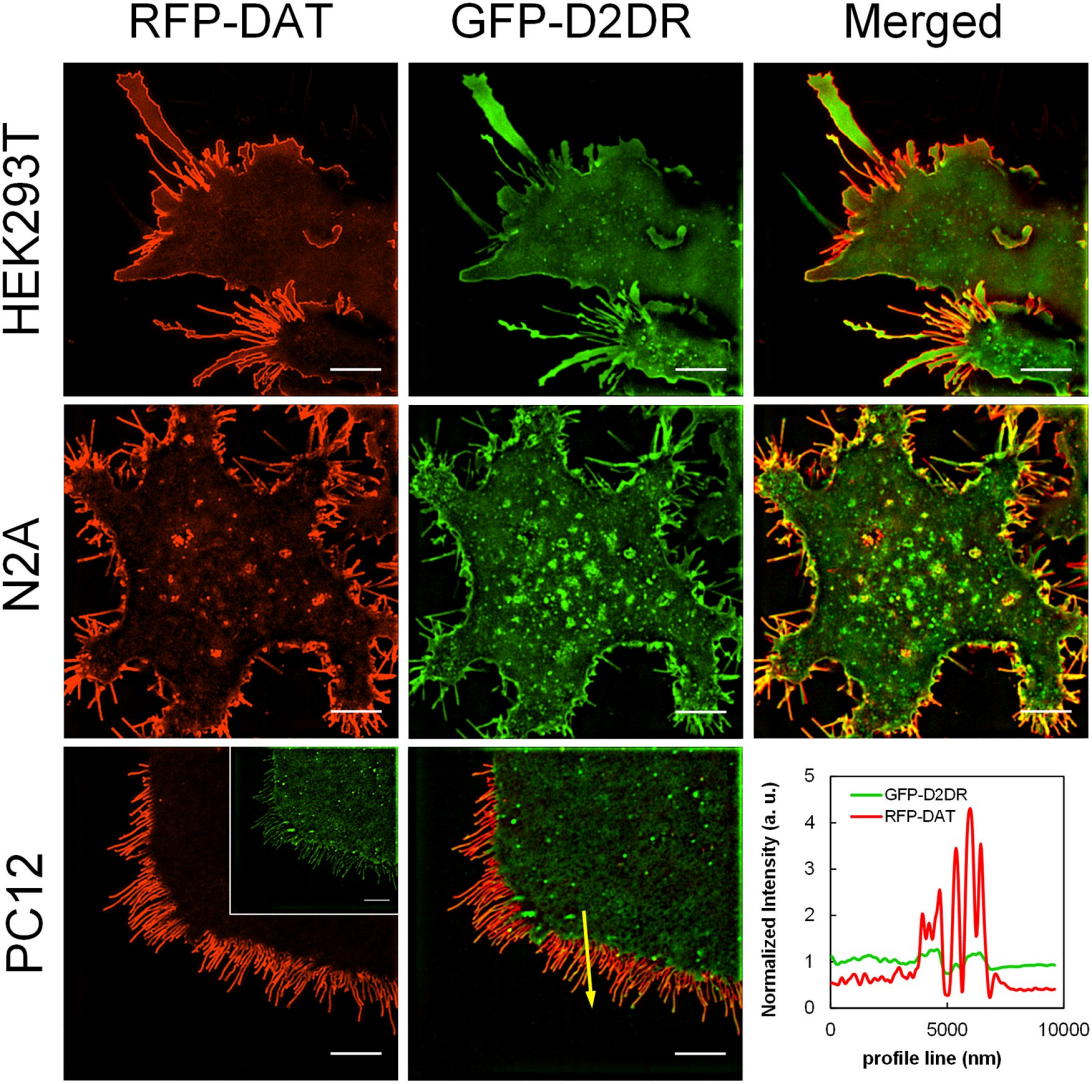
Differential membrane localization of RFP-DAT/GFP-D2DR in various cell hosts revealed via SIM superresolution microscopy. Bottom left: an image of the distribution of RFP-DAT expressed in a PC12 cell; inset is an image of the distribution of GFP-D2DR in the same cell. Bottom center: an image of RFP-DAT/GFP-D2DR colocalization in the PC12 cell shown in bottom left. Bottom right: the plot represents an intensity profile of the yellow line drawn across a segment of a membrane of a PC12 cells coexpressing RFP-DAT and GFP-D2DR. Scale bar: 5 μm. Images are representative of at three independent experiments. Two MatTek dishes plated per passage with three to six 512x512 fields of view acquired per MatTek dish constituted an independent experiment.

Next, we sought to rule out the possibility that the preferential targeting and retention of DAT-Qdots in the membrane edges/protrusions is merely an artifact of Qdot labeling. We examined the diffusion dynamics of a presynaptic protein partner of DAT, the D2 dopamine receptor (D2DR). Briefly, we labeled surface D2DR fused to the 3xHA epitope at the extracellular N-terminus with either biotinylated IDT772 [32] featuring modular architecture identical to that of IDT444 or a commercially available, high-affinity, biotinylated anti-HA antigen-binding fragment (Fab). Representative frames, 60-s maximum intensity projections, and reconstructed Qdot trajectories of the ligand- and anti-HA-Fab-tagged D2DRs are displayed in Fig 4a–4c (S6 File) and Fig 4d–4f (S7 File) respectively. A readily apparent uniform distribution of D2DR-Qdots indicated that the asymmetric surface distribution is independent of the targeting strategy and is specific for DAT. In parallel, we coexpressed RFP-fused DAT and GFP-fused D2DR in three heterologous cell hosts and imaged differences in DAT versus D2DR distribution via structured illumination microscopy (SIM), an advanced widefield microscopy technique that uses patterned illumination to double the lateral spatial resolution [48]. Fig 5 displays representative SIM images of RFP-DAT/GFP-D2DR distribution in HEK293T, N2A, and PC12 cells. In agreement with prior observations, RFP-DAT appeared to be preferentially accumulated at the cell periphery and in thin membrane protrusions, whereas GFP-D2DR signal was more uniformly localized over the entire cell surface. To sum up, DAT preferentially accumulated in the membrane edges/protrusions in all cell hosts examined, and DAT-Qdot diffusion was restricted from the flat membrane zone in a DAT-selective manner independently of the Qdot labeling strategy.

### Probing the role of membrane lipids and cytoskeleton in DAT spatiotemporal distribution

Next, we examined how manipulating the membrane lipid composition or disrupting the cytoskeleton affected preferential DAT targeting to the membrane edges/protrusions. It is an established fact that cholesterol, a component of membrane microdomains and a known modulator of DAT-mediated dopamine reuptake, interacts with multiple DAT transmembrane domains [49,50]. Phosphatidylinositol 4,5-bisphosphate (PIP_2_), a small phospholipid at the inner leaflet of the membrane, electrostatically engages the positively charged DAT N-terminus and modulates DAT conformational equilibrium [51]. The integrity of the cytoskeletal network comprised of actin and microtubules appears to be necessary to sustain constitutive DAT endocytic trafficking and may be required to stabilize cell surface DATs [52,53]. Therefore, we sought to determine the ratio of RFP-DAT-WT fluorescence intensity in edges/protrusions to that of RFP-DAT-WT in flat membrane regions in transfected HEK293T cells treated with 5 mM methyl-β-cyclodextrin for 30 min, 10 μM m-3M3FBS or its inactive analog o-3M3FBS for 10 min, 2.5 μM latrunculin B for 10 min, or 10 μM nocodazole for 1 hr to achieve cholesterol depletion, PIP_2_-phospholipid depletion, actin disruption, or microtubule depolymerization, respectively. A decrease in the ratio would indicate RFP-DAT shift away from the membrane edges and protrusions.

**Fig 6:**
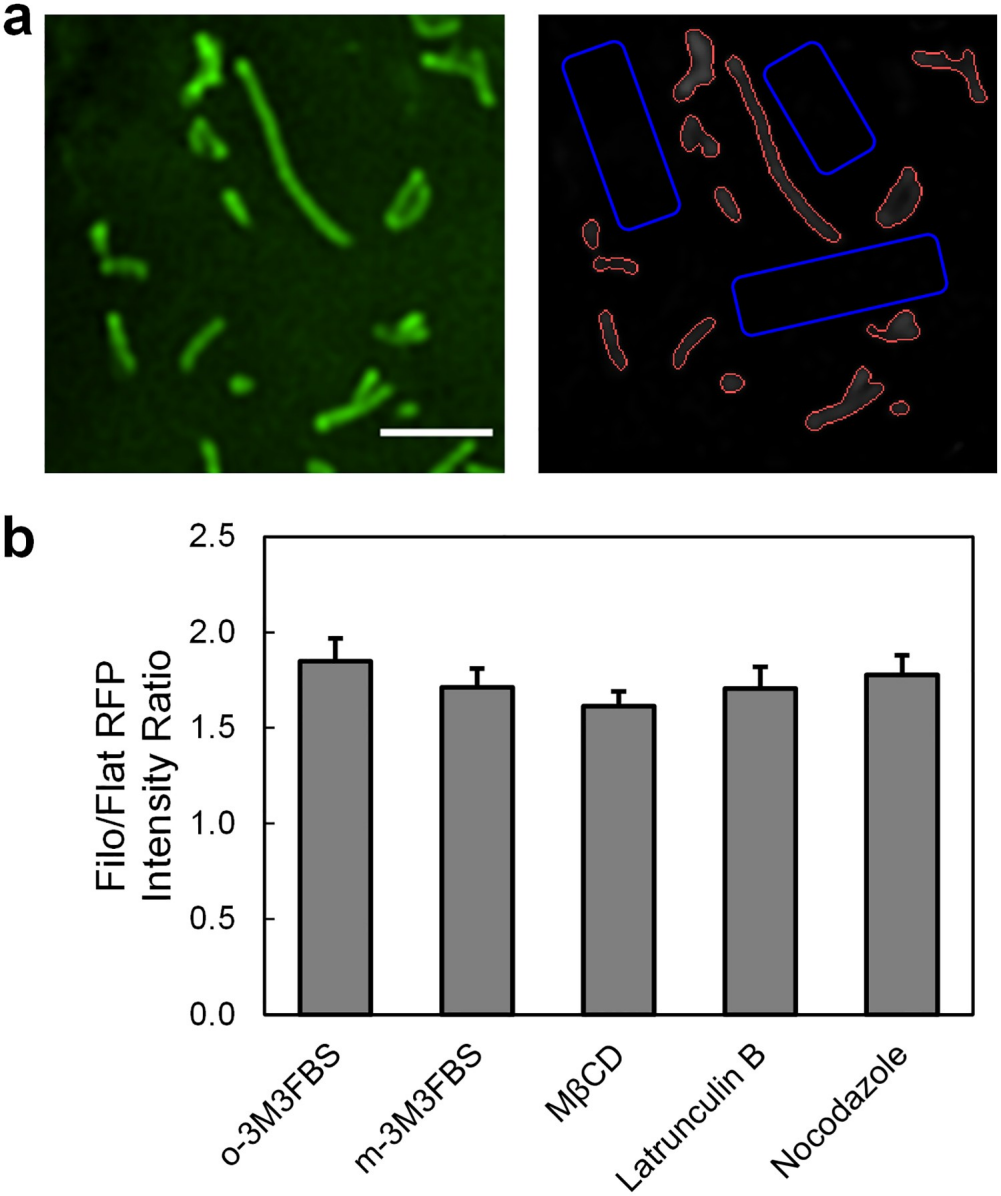
Lack of effect of cholesterol/PIP_2_ depletion and cytoskeleton disruption on asymmetric DAT membrane distribution. (a) Ratio of RFP-DAT intensity in the membrane edge/protrusions (red areas) to that in flat membrane regions (blue areas) was calculated for HWK-293T cells treated with o-3M3FBS (control), m-3M3FBS, MβCD, latrunculin B, and nocodazole. (b) A total of 9, 16, 11, 12 and 16 field of views were examined for each condition respectively in three independent experiments. o-3M3FBS (inactive analog of m-3M3FBS) was used as the control group. p>0.05 for all groups vs control, unpaired Student’s t-test.

To our surprise, these treatments did not have a significant effect on the asymmetric DAT membrane distribution (Fig 6). Although PIP_2_ was shown to interact with DAT N-terminus [51], we observed no significant effect of short-term PIP_2_ depletion via m-3M3FBS-mediated phospholipase C activation on DAT enrichment in the membrane edges/protrusions. Interestingly, our result is in agreement with the previously reported lack of effect of DAT N-terminal Δ36 deletion on DAT enrichment in filopodia [15], indicating that PIP_2_ does not appear to be responsible for DAT retention in the membrane edges/protrusions. We did however detect a significant increase in the diffusion rate (an average *D*_*MLE*_ increase of 17%) of the Qdot-tagged DAT in m-3M3FBS-treated HEK-293T cells compared to o-3M3FBS-treated control cells, suggesting that PIP_2_ may be involved in stabilizing DAT surface diffusion locally rather than maintaining separation of distinct DAT pools in the membrane edges/protrusions versus the flat membrane region (S3 and S4 Figs). More surprisingly, our data show that acute cholesterol depletion via MβCD did not result in RFP-DAT redistribution away from the membrane edges/protrusions, despite the importance of cholesterol in stabilizing DAT outward-facing conformation [49]. Hong and Amara showed that increasing membrane cholesterol in HEK-293 cells and striatal synaptosomes promoted cocaine binding to DAT and enhanced sulfhydryl accessibility of cysteine 306, a juxtamembrane residue on DAT TM6 that is a reliable sensor of the outward-facing DAT. It has been suggested that the cholesterol molecule wedged within a groove formed by DAT transmembrane helices 1a, 5 and 7 prevents its outward-to-inward transition [50,54]. Although MβCD is effective in depleting both raft and non-raft membrane cholesterol fractions, it is possible that the lack of response in our studies can be attributed to its poor ability to eliminate DAT-bound cholesterol molecule. Nevertheless, our data indicate that the level of membrane cholesterol is not required for DAT retention in the membrane edges/protrusions.

To achieve cytoskeleton disruption, we treated RFP-DAT-transfected HEK-293T cells with latrunculin B, a marine toxin widely used to depolymerize actin filament, and nocodazole, a small-molecule antimitotic agent that binds to β-tubulin and inhibits microtubule dynamics. The filopodia/edge-to-flat membrane region RFP-DAT intensity ratio was then measured in the treated HEK-293T cells. No significant difference was observed for either latrunculin B- or nocodazole-treated cells, indicating that DAT retention in the membrane edge/protrusions is cytoskeleton-independent. Additionally, these cytoskeleton-disrupting treatments did not significantly affect the rate of diffusion of surface DAT molecules (S3 and S4 Figs).

### Assessment of the effects of conformation-disrupting mutations on DAT diffusion dynamics

**Fig 7:**
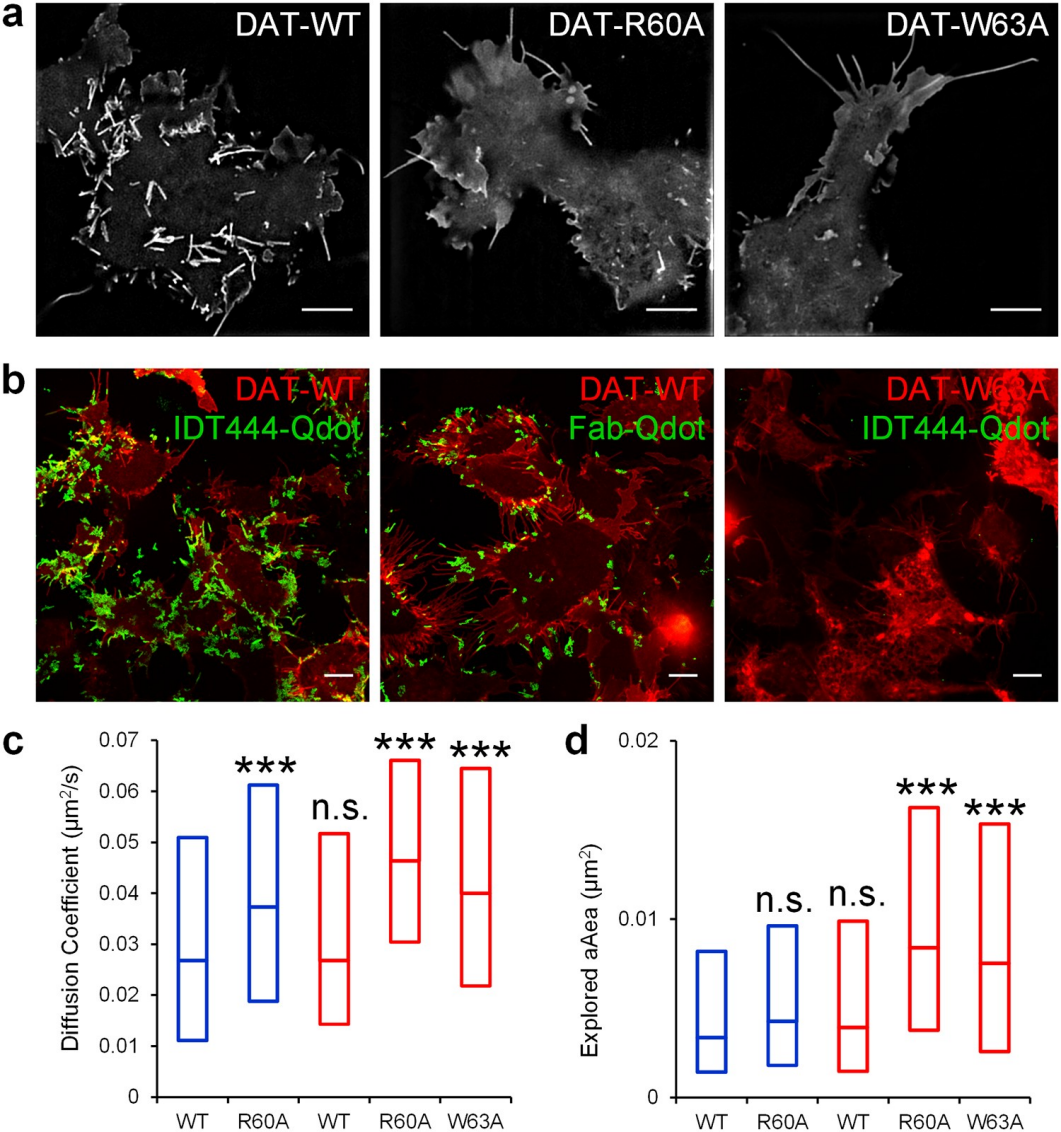
Effects of DAT conformation state on DAT membrane distribution and diffusion dynamics. (a) SIM superresolution images of RFP-HA-DAT, YFP-HA-DAT-R60A, and YFP-HA-DAT-W63A distribution. Scale bar: 5 μm. Images are representative of at least three independent experiments. Two MatTek dishes plated per passage with three to six 512x512 fields of view acquired per MatTek dish constituted an independent experiment. (b) Representative 60-s MIP trajectories of DAT-Qdots labeled either with biotinylated IDT444 (left) or anti-HA-biotin Fab (center) are shown. Note the lack of Qdot labeling of the dysfunctional, inward-facing W63A DAT variant (right). Scale bar: 10 μm. (c and d) Analysis of the diffusion dynamics of WT DAT and its R60A and W63A dysfunctional variants. For IDT444-labeled (blue) transporter: N_WT_ = 2861 tracks, N_R60A_ = 1480 tracks; for anti-HA-biotin-tagged (red) transporter: N_WT_ = 669 tracks, N_R60A_ = 445 tracks, N_W63A_ = 533 tracks. ***p<0.001, Kolmogorov-Smirnov test and one-way ANOVA with Dunnett’s post-hoc test. Median values and 25–75% interquartile ranges are tabulated in Table 2. Source data are provided in S2 Dataset.

Several recent reports from the Sorkin group [16–18] demonstrated that the outward-facing wildtype DAT and the derived DAT constructs (wildtype DAT with HA tag in EL2, N-terminal RFP-fused wildtype DAT, and N-terminal YFP-fused wildtype DAT) all favored localization to the highly curved membrane regions, such as filopodia. In contrast to the wildtype transporter, dysfunctional DAT mutants R60A and W63A with a disrupted outward-facing state did not concentrate in these membrane regions to the same extent and displayed a more uniform plasma membrane distribution. Indeed, when we transiently expressed RFP-HA-DAT-WT, YFP-HA-DAT-R60A, and YFP-HA-DAT-W63A in HEK-293T cells, we observed that the R60A and W63A substitution abolished preferential DAT accumulation in the membrane protrusions as evidenced by the representative SIM images (Fig 7a).

Next, we implemented our SPT experimental framework to assess the effects of the R60A and W63A substitution on the membrane diffusion dynamics of DAT. In line with the decreased rate of [^3^H]dopamine uptake inward-facing transporter variants [16], we observed reduced labeling efficiency of DAT R60A with antagonist-conjugated Qdots and were unable to label the completely non-functional, inward-facing W63A transporter mutant with antagonist-conjugated Qdots (Fig 7b). In another set of SPT experiments, we utilized an offsite labeling strategy, which did not require the availability of the DAT substrate binding site. The offsite strategy utilized the biotinylated anti-HA-Fab targeting the HA epitope incorporated into the EL2 of the three DAT variants and allowed us to tag the W63A variant with Qdots [18]. Interestingly, we saw considerably reduced Qdot labeling efficiency of DAT using anti-HA-Fab compared to the IDT444 antagonist (Fig 7b). This finding is in line with the previous observation by Wu et al. [38] that both the HA and the unflanked LAP epitopes in the DAT EL2 suffer from poor labeling efficiency by anti-HA antibody and alkyne-Alexa Fluor respectively, likely due to the hindered access as DAT possesses a compact 3D structure. We then recorded lateral motion of Qdot-tagged DAT variants over 60 s and compared the diffusion rate (*D*_*MLE*_) of the mobile fractions (Fig 7c; Table 2; S5 Fig). Lateral mobility for the R60A transporter variant labeled with IDT444-Qdots increased significantly compared to the WT protein (Fig 7c; Table 2).

**Table 2:** Conformation-dependent changes in DAT diffusion dynamics.

DAT Variant	Probe	Median *D*_*MLE*_ [IQR]	Explored Area [IQR]	N	Mann-Whitney U-test	K-S test
WT	IDT444	0.027 μm^2^/s [0.011–0.051]	0.0033 μm^2^ [0.0014–0.0082]	2861	-----	-----
R60A		0.037 μm^2^/s [0.019–0.061]	0.0043 μm^2^ [0.0018–0.0096]	1480	D: vs WT/IDT444: p = 3x10^-23^ E.A.: vs WT/IDT444: p = 4x10^-7^	D: vs WT/IDT444: p = 6x10^-20^ E.A.: vs WT/IDT444: p = 3x10^-7^
WT	Anti-HA Fab	0.027 μm^2^/s [0.014–0.052]	0.0039 μm^2^ [0.0015–0.0099]	669	D: vs WT/IDT444: p = 0.06 E.A.: vs WT/IDT444: p = 0.0069	D: vs WT/IDT444: p = 0.0017 E.A.: vs WT/IDT444: p = 0.0004
R60A		0.046 μm^2^/s [0.030–0.066]	0.0084 μm^2^ [0.0038–0.0163]	445	D: vs WT/Fab: p = 5x10^-20^ D: vs R60A/IDT444: p = 3x10^-7^ E.A.: vs WT/Fab: p = 6x10^-17^ E.A.: vs R60A/IDT444: p = 7x10^-20^	D: vs WT/Fab: p = 7x10^-21^ D: vs R60A/IDT444: p = 1x10^-7^ E.A.: vs WT/Fab: p = 5x10^-17^ E.A.: vs R60A/IDT444: p = 6x10^-18^
W63A		0.040 μm^2^/s [0.022–0.065]	0.0075 μm^2^ [0.0026–0.0153]	633	D: vs WT/Fab: p = 2x10^-9^ E.A.: vs WT/Fab: p = 2x10^-9^	D: vs WT/Fab: p = 3x10^-9^ E.A.: vs WT/Fab: p = 4x10^-9^

When tagged with anti-HA-Fab-Qdots, lateral mobility also increased significantly for both the R60A and W63A dysfunctional variants compared to the WT transporter (Table 2). Notably, the diffusion rate of the WT transporter was not dependent on the labeling approach in our experiments (Table 2). Explored area, computed as the area of the convex hull over the entire trajectory segment normalized to the number of time points and indicative of the diffusive behavior over the entire recording period, was also significantly higher for the dysfunctional R60A and W63A transporters when tagged with anti-HA-Fab-Qdots (Fig 7d, Table 2). However, we did not detect a significant difference between the explored areas of the IDT444-Qdot-labeled WT and R60A transporters (Fig 7d, Table 2). This finding might be explained in part by the propensity of cocaine and its analogs to stabilize the outward-facing DAT state and thereby influence the plasma membrane distribution of the dysfunctional R60A variant (Ma et al., 2017). Overall, it appears that the disruption of the amino acids within a conserved intracellular interaction network that modulates DAT conformation profoundly influences DAT plasma membrane distribution and affects the population diffusion dynamics of DAT proteins at the single particle level.

## Discussion

In this study, we used antagonist-conjugated Qdots to systematically characterize the cell surface diffusion dynamics of DATs in several heterologous expression systems. We observed a large degree of heterogeneity in the diffusion dynamics of dopamine transporters. The application of the relative deviation analysis to time-averaged MSD plots allowed us to identify distinct diffusive pools of cell surface DATs, with the majority of DATs exhibiting unrestricted, Brownian motion and a considerable fraction of DATs displaying restricted diffusion as shown in Fig 2. The existence of distinct diffusive pools of membrane DATs is expected according to the predominant hypothesis of DAT plasma membrane organization reported in the literature. It proposes a lipid microdomain model, suggesting that DAT segregates into cholesterol- and glycosphingolipid-rich nanodomains that provide the necessary microenvironment for efficient dopamine reuptake and potentially serve as “hot spots” for the recruitment of DAT binding partners or constitutive/regulated endocytosis [11–13,49,55]. When we examined factors involved in the regulation of DAT diffusion dynamics and cell surface distribution, we found that the lateral motion of DAT was primarily regulated through two mechanisms: (i) confinement and retention of a large fraction of DATs at the cell edge and (ii) limited diffusive DAT penetration into the flat membrane regions despite the existence of a highly mobile fraction in thin membrane protrusions.

The plasma membrane of DAT-expressing HEK-293T cells at the cell-coverslip interface possessed three prominent membrane features which most Qdot-tagged DATs were localized to–thin, finger-like protrusions (filopodia), thin flat membrane sheets (lamellipodia), and the cell edge. We correlated DAT diffusivity with the local membrane landscape by extracting two motion parameters, *D*_*MLE*_ and *α*, from the trajectories in the three membrane regions and found a striking dependence of DAT lateral mobility on its membrane location. Qdot-tagged DATs localized to the cell edge were moving laterally at a significantly slower rate in a subdiffusive regime than Qdot-tagged DATs found in membrane protrusions or lamellipodia (Fig 2c and 2d). Interestingly, the latter membrane areas are zones of active actin cytoskeleton remodeling, which has been shown to transiently organize biochemical reactions through active nanoclustering of various surface molecules and thereby spatiotemporally orchestrate cellular signaling [56].

**Fig 8:**
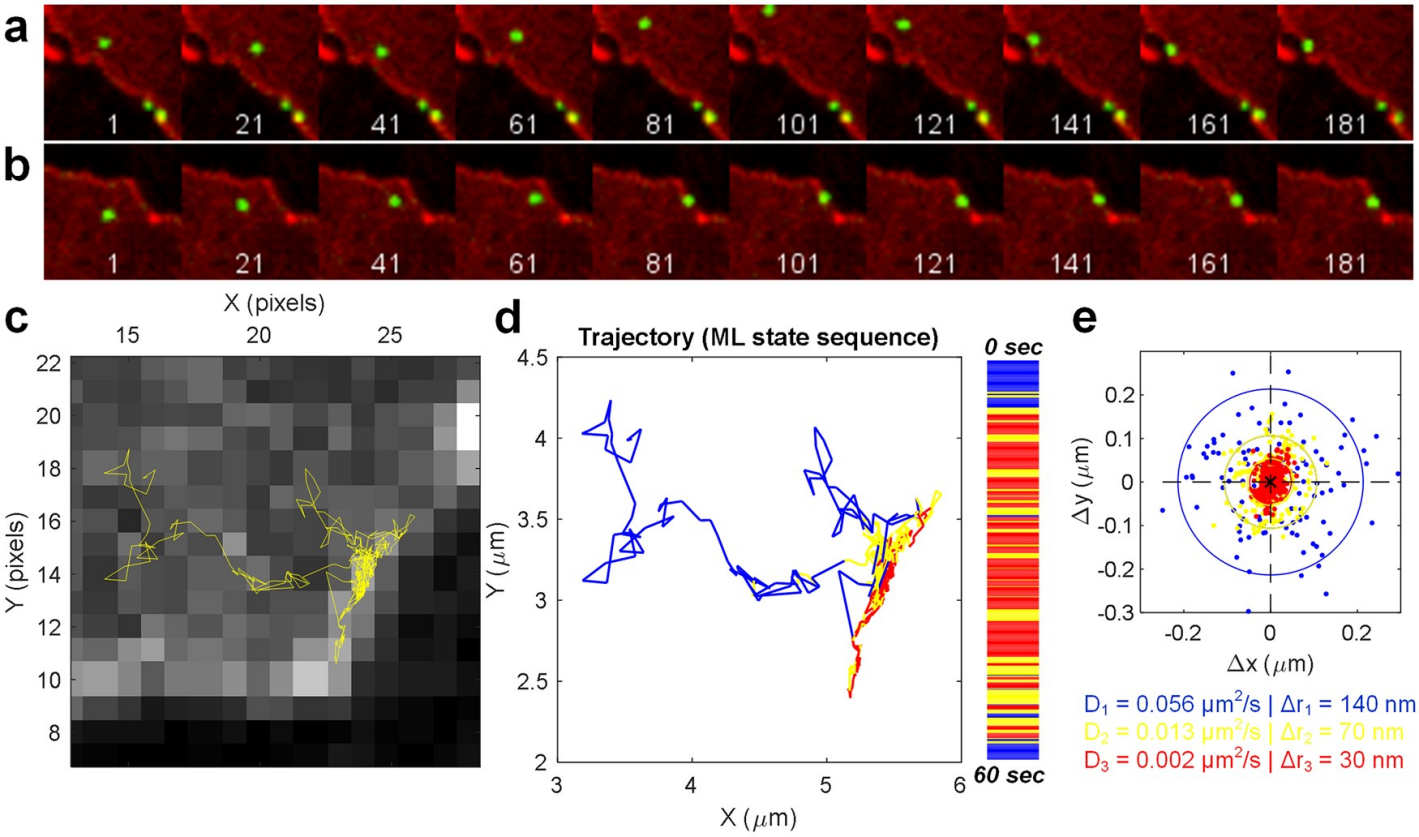
Dynamic retention of diffusing DAT-Qdots at the cell edge, an area of local DAT accumulation. (a-b) Representative time-lapse sequences showing transient trapping of DAT-Qdots at the cell edge, an area with increased local RFP-DAT density. Area size: 13x13 μm. (c)A reconstructed trajectory depicting DAT-Qdot motion in b. Scale: 1 pixel = 0.22 μm. (d) HMM Analysis of DAT-Qdot diffusive state swtiching over the course of the trajectory shown in c. Three distinct diffusive states were identified–fast (blue) occurring in the flat membrane regions, yellow and red (intermediate and slow) occurring in the vicinity of the cell edge and corresponding to transient DAT-Qdot trapping. (e) A scatter plot showing the distribution of instantaneous vector displacements (Δx,Δy) corresponding to the three diffusive states identified in d. Source data are provided in S3 Dataset.

Monitoring surface DAT-Qdots on a longer time scale (30+ min) revealed persistent avoidance of the flat membrane regions in all cell types examined. We ruled out the possibility that such asymmetric spatiotemporal distribution of surface DAT-Qdot localizations is artifactually caused by Qdot targeting through the application of our labeling strategy to the D2 dopamine receptor. The uniform plasma membrane distribution of both ligand-occupied and anti-HA-Fab-tagged Qdot-D2DRs was readily apparent in contrast to the DAT-Qdot localization pattern. We confirmed our Qdot-based findings by employing an orthogonal imaging modality, superresolution SIM microscopy to visualize differential membrane distribution of RFP-tagged DAT and GFP-tagged D2DR coexpressed in various cell hosts. The striking polarization of DAT surface density was independent of the labeling strategy and in agreement with the reports by Sorkin and colleagues on DAT preferential accumulation in filopodia [17,18]. Although the flat membrane regions of cells we imaged were usually devoid of DAT-Qdots, occasionally we were able to capture DAT-Qdots diffusing in the flat membrane region in the vicinity of the cell edge (Fig 8, S8 and S9 Files). Once DAT-Qdots penetrated into the cell edge region with higher DAT density, they underwent transient trapping when diffusing into the DAT-rich membrane edge.

Several lines of evidence have recently demonstrated that the outward-facing conformation of DAT is necessary for its accumulation in the filopodia of nonneuronal and neuronal cells [16–18]. Specifically, the Ala substitution of Arg60 (R60) and Trp63 (W63) amino acid residues, which are necessary for DAT to adopt the outward-facing conformation, abolishes preferential DAT concentration in filopodia. We confirmed this phenomenon via superresolution SIM microscopy and then analyzed the relative diffusion rates of DAT-WT-Qdots, DAT-R60A-Qdots, and DAT-W63A-Qdots at the plasma membrane of HEK293T cells. We showed that both DAT variants with the disrupted outward-facing conformation were diffusing at a significantly faster rate at the cell surface. Importantly, this difference in mobility was consistent for both antibody- and ligand-bound transporters and was well correlated with acute changes in the polarization of DAT surface density. Since DAT sorting into the membrane edges/protrusions does not appear to rely on a cytoskeleton- or lipid-dependent mechanism, we suggest that the exclusion of DAT from the flat membrane regions is instead mediated via biomechanical coupling between the intrinsic DAT curved shape (the diameter of the cytoplasmic interface of the DAT core is smaller than that of the extracellular interface [54] and the membrane curvature [57]. Recently, Rosholm and colleagues [57] proposed and experimentally validated that the retention of the asymmetric neuropeptide Y receptor Y2 (Y2R) in the curved membrane protrusions of various cell lines is a result of the balance between the energetic drive to match membrane and protein curvature and the entropic resistance of having different protein densities in the protrusion and the membrane reservoir. In contrast, no curvature-mediated sorting was observed for the uniform, cylindrical aquaporin 0 (AQP0) water channel. A direct outcome of this hypothesis is that curvature-dependent sorting of a transmembrane protein should in principle be sensitive to its conformational state. In fact, the introduction of R60A and W63A mutations into DAT structure that bias the transporter to an inward-facing state essentially eliminate preferential DAT targeting to the membrane edges/protrusions. The profound impact of the point mutations that bias the transporter to the inward-facing state on the spatiotemporal membrane organization of DAT has particularly important implications for psychiatric disorders associated with rare DAT coding variants. Investigating the precise mechanisms that govern DAT diffusion dynamics and its surface density polarization in healthy versus disease models may thus lead to a better understanding of the molecular pathology of these brain disorders.

## Conclusions

We conducted a systematic study characterizing the diffusive properties of the cell surface DAT molecules using our SPT framework based on antagonist-conjugated Qdots. SPT and superresolution SIM experiments revealed that DAT surface density is highly polarized and DAT membrane diffusivity is strongly dependent on the membrane features DAT resides in–membrane edge, protrusions, lamellipodia, or flat membrane zones. We show that the exclusion of DAT from the flat membrane regions is not a lipid- or cytoskeleton-dependent phenomenon and propose that it is instead mediated via biomechanical coupling between the intrinsic DAT curved shape and the high curvature of various plasma membrane feature (edge, protrusions, kinks, grooves, invaginations). We also show that the surface dynamics of DAT molecules are highly sensitive to the conformational state of the transporter. Together, these biophysical phenomena warrant an in-depth investigation as to whether compromised surface diffusion is a common pathological feature of brain disorder-derived DAT variants and how it impacts the efficiency of dopamine neurotransmission *in vivo*.

## Supporting information

S1 Dataset Source data for Fig 2. (XLSX)

S2 Dataset Source data for Fig 7. (XLSX)

S3 Dataset Source data for Fig 8—HMM-Bayes analysis output from Matlab. (ZIP)

S1 Fig Relative deviation analysis of individual simulated trajectories. Histograms of trajectory number for deduced RD(*N*,25) for 1,000 simulated Brownian (free/nonanomalous) trajectories with varying track length *N* = 100, 200, 300, 400, and 600.
(TIF)

S2 Fig RD(*N*,25) versus *track length (N)* for simulations in S1 Fig. (a) Solid circles indicate 2.5th and 97.5th percentiles. (b) Computed fractions of simulated trajectories (each containing 1,000 tracks of varying length, i.e. 50–600 frames) classified as undergoing free diffusion (white) versus restricted diffusion (gray).
(TIF)

S3 Fig Diffusion rate of RFP-DAT-WT in transiently transfected HEK-293T cells under different conditions. Trajectories of Qdot-tagged RFP-DAT-WT were recorded a spinning disk confocal microscope at 10 Hz for 1 minute (a total of 2767 (o-3MF3BS), 3932 (m-3M3FBS), 3311 (Latrunculin B), 3173 (Nocodazole), 924 (apical membrane), and 3867 (cold imaging buffer) tracks ≥50 frames were analyzed from three independent experiments for each conditions. Differences between Dmle distributions for each condition versus the control group were tested for statistical significance using the Kolmogorov-Smirnov test (*** denotes p<0.001, ** denotes p<0.01).
(TIF)

S4 Fig Dot density plots depicting diffusion coefficient distributions shown in S3 Fig. The median is represented by the black line.
(TIF)

S5 Fig Dot density plots depicting diffusion coefficient distributions for IDT444- or anti-HA-Fab-tagged DAT variants. Median is represented by the black line.
(TIF)

S1 File Video of diffusing Qdot-tagged wildtype DAT in live HEK-293 cells. The time-lapse series were acquired at 10 Hz for 1 minute. The rate of the video playback is 30 frames/s.
(AVI)

S2 File Video of diffusing Qdot-tagged wildtype DAT in live N2A cells. The time-lapse series were acquired at 10 Hz for 1 minute. The rate of the video playback is 30 frames/s.
(AVI)

S3 File Longer-term video of diffusing Qdot-tagged wildtype DAT in live N2A cells. The time-lapse series were acquired at 0.1 Hz for 30 minutes. The rate of the video playback is 30 frames/s.
(AVI)

S4 File Video of diffusing Qdot-tagged wildtype DAT along a neurite in live N2A cells. The time-lapse series were acquired at 10 Hz for 1 minute. The rate of the video playback is 30 frames/s.
(AVI)

S5 File Longer-term video of diffusing Qdot-tagged wildtype DAT along a neurite in live N2A cells. The time-lapse series were acquired at 0.1 Hz for 15 minutes. The rate of the video playback is 30 frames/s.
(AVI)

S6 File Video of diffusing Qdot-tagged D2DR-HA in live HEK-293T cells. The time-lapse series were acquired at 33 Hz for 1 minute. The rate of the video playback is 30 frames/s. Surface D2DR-HA were sequentially labeled with the biotinylated IDT772 (500 nM) and Qdot605Sav (0.1 nM) for 10 and 5 minutes respectively.
(AVI)

S7 File Video of diffusing Qdot-tagged D2DR-HA in live HEK-293T cells. The time-lapse series were acquired at 33 Hz for 1 minute. The rate of the video playback is 30 frames/s. Surface D2DR-HA were sequentially labeled with the biotinylated anti-HA Fab (0.2 μg/mL) and Qdot605Sav (0.1 nM) for 10 and 5 minutes respectively.
(AVI)

S8 File Video of transient trapping of Qdot-tagged RFP-HA-DAT at the cell edge in live HEK293T cells. The time-lapse series were acquired at 10 Hz for 1 minute. The rate of the video playback is 30 frames/s.
(AVI)

S9 File Video of transient trapping of Qdot-tagged RFP-HA-DAT at the cell edge in live HEK293T cells. The time-lapse series were acquired at 10 Hz for 1 minute. The rate of the video playback is 30 frames/s.
(AVI)

## References

[1] Giros B, Giros B, Caron MG. Molecular characterization of the dopamine transporter. Trends Pharmacol Sci. 1993; 14: 43–49. 10.1016/0165-6147(93)90029-j 8480373

[2] Girault J-A, Greengard P. The neurobiology of dopamine signaling. Arch Neurol. 2004; 61: 641–644. 10.1001/archneur.61.5.641 15148138

[3] Gether U, Andersen PH, Larsson OM, Schousboe A. Neurotransmitter transporters: molecular function of important drug targets. Trends in Pharmacol Sci. 2006; 27: 375–383. 16762425 10.1016/j.tips.2006.05.003

[4] Torres GE, Gainetdinov RR, Caron MG. Plasma membrane monoamine transporters: structure, regulation and function. Nature Rev Neurosci. 2003; 4: 13–25. 12511858 10.1038/nrn1008

[5] Greenwood TA, Alexander M, Keck PE, McElroy S, Sadovnick AD, Remick RA, et al Evidence for linkage disequilibrium between the dopamine transporter and bipolar disorder. Am J Med Genet. 2001; 105: 145–151. 10.1002/1096-8628(2001)9999:9999<::aid-ajmg1161>3.0.co;2-8 11304827

[6] Swanson JM. Dopamine genes and ADHD. Neurosci Biobehav Rev. 2000; 24: 21–25. 10.1016/s0149-7634(99)00062-7 10654656

[7] Kirley A, Lowe N, Hawi Z, Mullins C, Daly G, Waldman I, et al Association of the 480 bp DAT1 allele with methylphenidate response in a sample of Irish children with ADHD. Am J Med Genet Part B. 2003; 121B: 50–54. 10.1002/ajmg.b.20071 12898575

[8] Kurian MA, Zhen J, Cheng SY, Li Y, Mordekar SR, Jardine P, et al Homozygous loss-of-function mutations in the gene encoding the dopamine transporter are associated with infantile parkinsonism-dystonia. J Clin Invest. 2009; 119: 1595–1603. 10.1172/JCI39060 19478460 PMC2689114

[9] Gowrishankar R, Hahn MK, Blakely RD. Good riddance to dopamine: roles for the dopamine transporter in synaptic function and dopamine-associated brain disorders. Neurochem Int. 2014; 73: 42–48. 10.1016/j.neuint.2013.10.016 24231471

[10] Kaya C, Cheng MH, Block ER, Bartol TM, Sejnowski TJ, Sorkin A, et al Heterogeneities in axonal structure and transporter distribution lower dopamine reuptake efficiency. eNeuro 2018; 5: e0298-17.2017, 1–21. 10.1523/ENEURO.0298-17.2017 PMC5804147 29430519

[11] Adkins EM, Samuvel DJ, Fog JU, Eriksen J, Jayanthi LD, Vaegter CB, et al Membrane mobility and microdomain association of the dopamine transporter studied with fluorescence correlation spectroscopy and fluorescence recovery after photobleaching. Biochemistry. 2017; 46: 10484–10497. 10.1021/bi700429z 17711354

[12] Eriksen J, Jorgensen TN, Gether U. Regulation of dopamine transporter function by protein-protein interactions: new discoveries and methodological challenges. J Neurochem. 2010; 113: 27–41. 10.1111/j.1471-4159.2010.06599.x 20085610

[13] Cremona ML, Matthies HJ, Pau K, Bowton E, Speed N, Lute BJ, et al Flotillin-1 is essential for PKC-triggered endocytosis and membrane microdomain localization of DAT. Nature Neuroscience. 2011; 14: 469–477. 10.1038/nn.2781 21399631 PMC3066276

[14] Eriksen J, Rasmussen SG, Rasmussen TN, Vaegter CB, Cha JH, Zou MF, et al Visualization of dopamine transporter trafficking in live neurons by use of fluorescent cocaine analogs. J Neurosci, 2009; 29: 6794–6808. 10.1523/JNEUROSCI.4177-08.2009 19474307 PMC3849467

[15] Rahbek-Clemmensen T, Lycas MD, Erlendsson S, Eriksen J, Apuschkin M, Vilhardt F, et al Super-resolution microscopy reveals functional organization of dopamine transporters into cholesterol and neuronal activity-dependent nanodomains. Nat Commun. 2017; 8: 740 10.1038/s41467-017-00790-3 28963530 PMC5622129

[16] Sorkina T, Richards TL, Rao A, Zahniser NR, Sorkin A. Negative regulation of dopamine transporter endocytosis by membrane-proximal N-terminal residues. J Neurosci. 2009; 29: 1361–1374. 10.1523/JNEUROSCI.3250-08.2009 19193883 PMC2745124

[17] Caltagarone J, Ma S, Sorkin A. Dopamine transporter is enriched in filopodia and induces filopodia formation. Mol Cell Neurosci. 2015; 68: 120–130. 10.1016/j.mcn.2015.04.005 25936602 PMC4593718

[18] Ma S, Cheng MH, Guthrie DA, Newman AH, Bahar I, Sorkin A. Targeting of dopamine transporter to filopodia requires an outward-facing conformation of the transporter. Sci Rep. 2017; 14:5339. 10.1038/s41598-017-05637-x PMC5511133 28710426

[19] Sakrikar D, Mazei-Robison MS, Mergy MA, Richtand NW, Han Q, Hamilton PJ, et al Attention deficit/hyperactivity disorder-derived coding variation in the dopamine transporter disrupts microdomain targeting and trafficking regulation. J Neurosci. 2012; 32: 5385–5397. 10.1523/JNEUROSCI.6033-11.2012 22514303 PMC3342037

[20] Kovtun O, Tomlinson ID, Sakrikar DS, Chang JC, Blakely RD, Rosenthal SJ. Visualization of the cocaine-sensitive dopamine transporter with ligand-conjugated quantum dots. ACS Chem Neurosci. 2011; 2: 370–378. 10.1021/cn200032r 22816024 PMC3369746

[21] Kovtun O, Sakrikar D, Tomlinson ID, Chang JC, Arzeta-Ferrer X, Blakely RD, et al Single-quantum-dot tracking reveals altered membrane dynamics of an attention-deficit/hyperactivity-disorder-derived dopamine transporter coding variant. ACS Chem Neurosci. 2015; 6: 526–534. 10.1021/cn500202c 25747272 PMC5530757

[22] Rosenthal SJ, Chang JC, Kovtun O, McBride JR, Tomlinson ID. Biocompatible quantum dots for biological applications. Chem Biol. 2011; 18: 10–24. 10.1016/j.chembiol.2010.11.013 21276935 PMC3752999

[23] Chang JC, Tomlinson ID, Warnement MR, Ustione A, Carneiro AM, Piston DW, et al Single molecule analysis of serotonin transporter regulation using antagonist-conjugated quantum dots reveals restricted, p38 MAPK-dependent mobilization underlying uptake activation. J Neurosci. 2012; 32: 8919–8929. 10.1523/JNEUROSCI.0048-12.2012 22745492 PMC3426861

[24] Pinaud F, Clarke S, Sittner A, Dahan M. Probing cellular events, one quantum dot at a time. Nat Methods. 2010; 7: 275–285. 10.1038/nmeth.1444 20354518

[25] Kovtun O, Tomlinson ID, Bailey DM, Thal LB, Ross EJ, Harris L, et al Single quantum dot tracking illuminates neuroscience at the nanoscale. Chem Phys Lett. 2018; 706: 741–752. 10.1016/j.cplett.2018.06.019 30270931 PMC6157616

[26] Bailey DM, Catron MA, Kovtun O, Macdonald RL, Zhang Q, Rosenthal SJ. Single quantum dot tracking reveals serotonin transporter diffusion dynamics are correlated with cholesterol-sensitive threonine 276 phosphorylation status in primary midbrain neurons. ACS Chem Neurosci. 2018; 9:2534–2541. 10.1021/acschemneuro.8b00214 29787674

[27] Mason JN, Farmer H, Tomlinson ID, Schwartz JW, Savchenko V, DeFelice LJ, et al Novel fluorescence-based approaches for the study of biogenic amine transporter localization, activity, and regulation. J Neurosci Methods. 2005; 143: 3–25. 10.1016/j.jneumeth.2004.09.028 15763132

[28] Orndorff RL, Rosenthal SJ. Neurotoxin quantum dot conjugates detect endogenous targets expressed in live cancer cells. Nano Lett. 2009; 9: 2589–2599. 10.1021/nl900789e 19507837

[29] Rosenthal SJ, Tomlinson I, Adkins EM, Schroeter S, Adams S, Swafford L, et al Targeting cell surface receptors with ligand-conjugated nanocrystals. J Am Chem Soc. 2002; 124: 4586–4594. 10.1021/ja003486s 11971705

[30] Gussin HA, Tomlinson ID, Little DM, Warnement MR, Qian H, Rosenthal SJ, et al Binding of muscimol-conjugated quantum dots to GABAC receptors. J Am Chem Soc. 2006; 128: 15701–15713. 10.1021/ja064324k 17147380 PMC2553244

[31] Gussin HA, Tomlinson ID, Cao D, Qian H, Rosenthal SJ, Pepperberg DR. Quantum dot conjugates of GABA and muscimol: binding to α1β2γ2 and ρ1 GABA(A) receptors. ACS Chem Neurosci. 2013; 4: 435–443. 10.1021/cn300144v 23509979 PMC3605815

[32] Tomlinson ID, Kovtun O, Rosenthal SJ. Biotinylated-spiperone ligands for quantum dot labeling of the Dopamine D2 receptor (D2 DR) in live cell cultures. Bioorg Med Chem Lett. 2019; 29:959–964. 10.1016/j.bmcl.2019.02.024 30808590

[33] Jaqaman K, Loerke D, Mettlen M, Kuwata H, Grinstein S, Schmid SL et al Robust single particle tracking in live cell time-lapse sequences. Nat Methods. 2008; 5: 695–702. 10.1038/nmeth.1237 18641657 PMC2747604

[34] Michalet X, Berglund AJ. Optimal diffusion coefficient estimation in single-particle tracking. Phys Rev E Stat Nonlin Soft Matter Phys. 2010; 85: 061916. 10.1103/PhysRevE.85.061916 PMC4917385 23005136

[35] Kusumi A, Sako Y, Yamamoto M. Confined lateral diffusion of membrane receptors as studied by single particle tracking (nanovid microscopy). Effects of calcium-induced differentiation in cultured epithelial cells. Biophys J. 1993; 65: 2021–2040. 10.1016/S0006-3495(93)81253-0 8298032 PMC1225938

[36] Crane JM, Verkman AS. Long-range nonanomalous diffusion of quantum dot-labeled aquaporin-1 water channels in the cell plasma membrane. Biophys J. 2008; 94: 702–713. 10.1529/biophysj.107.115121 17890385 PMC2157255

[37] Monnier N, Barry Z, Park HY, Su KC, Katz Z, English BP, et al Inferring transient particle transport dynamics in live cells. Nat Methods. 2015; 12: 838–840. 10.1038/nmeth.3483 26192083 PMC4733533

[38] Wu S, Fagan RR, Uttamapinant C, Lifshitz LM, Fogarty KE, Ting AY, et al The dopamine transporter recycles via a retromer-dependent postendocytic mechanism: tracking studies using a novel fluorophore-coupling approach. J Neurosci. 2017; 37: 9438–9452. 10.1523/JNEUROSCI.3885-16.2017 28847807 PMC5618262

[39] Murphy-Royal C, Dupuis JP, Varela JA, Panatier A, Pinson B, Baufreton J, et al Surface diffusion of astrocytic glutamate transporters shapes synaptic transmission. Nat Neurosci. 2015; 18: 219–226. 10.1038/nn.3901 25581361

[40] Vuorenpää A, Jørgensen TN, Newman AH, Madsen KL, Scheinin M, Gether U. Differential internalization rates and postendocytic sorting of the norepinephrine and dopamine transporters are controlled by structural elements in the N termini. J Biol Chem. 2016; 291: 5634–5651. 10.1074/jbc.M115.702050 26786096 PMC4786704

[41] Nirenberg MJ, Chan J, Vaughan RA, Uhl GR, Kuhar MJ, Pickel VM. Immunogold localization of the dopamine transporter: an ultrastructural study of the rat ventral tegmental area. J Neurosci. 1997; 17: 5255–5262. 10.1523/JNEUROSCI.17-14-05255.1997 9204909 PMC6793826

[42] Winckler B, Forscher P, Mellman I. A diffusion barrier maintains distribution of membrane proteins in polarized neurons. Nature. 1999; 397: 698–701. 10.1038/17806 10067893

[43] Cowan AE, Myles DG, Koppel DE. Lateral diffusion of the PH-20 protein on huinea pig sperm: Evidence that barriers to diffusion maintain plasma membrane domains in mammalian sperm. J Cell Biol. 1987; 104: 917–923. 10.1083/jcb.104.4.917 3558486 PMC2114437

[44] Rasband MN. The axon initial segment and the maintenance of neuronal polarity. 2010; 11: 552–562. 10.1038/nrn2852 20631711

[45] Peter BJ, Kent HM, Mills IG, Vallis Y, Butler PJ, Evans PR, et al BAR domains as sensors of membrane curvature: the amphiphysin BAR structure. Science. 2004; 303: 495–499. 10.1126/science.1092586 14645856

[46] Arizono M, Bannai H, Nakamura K, Niwa F, Enomoto M, Matsu-Ura T, et al Receptor-selective diffusion barrier enhances sensitivity of astrocytic processes to metabotropic glutamate receptor stimulation. Sci Signal 2012; 5: ra27 10.1126/scisignal.2002498 22472649

[47] Singer SJ, Nicolson GL. fluid mosaic model of the structure of cell membranes. Science. 1972; 175: 720–731. 10.1126/science.175.4023.720 4333397

[48] Gustafsson MG. Surpassing the lateral resolution limit by a factor of two using structured illumination microscopy. J Microsc. 2000; 198: 82–87. 10.1046/j.1365-2818.2000.00710.x 10810003

[49] Hong WC, Amara SG. Membrane cholesterol modulates the outward facing conformation of the dopamine transporter and alters cocaine binding. J Biol Chem. 2010; 285: 32616–32626. 10.1074/jbc.M110.150565 20688912 PMC2952264

[50] Zeppelin T, Laderfoged LK, Sinning S, Periole X, Schiøtt B. A direct interaction of cholesterol with the dopamine transporter prevents its out-to-inward transition. PLoS Comput Biol. 2018; 14:e1005907 10.1371/journal.pcbi.1005907 29329285 PMC5811071

[51] Hamilton PJ, Belovich AN, Khelashvili G, Saunders C, Erreger K, Javitch JA, et al PIP2 regulates psychostimulant behaviors through its interaction with a membrane protein. Nat Chem Biol. 2014; 10:582–589. 10.1038/nchembio.1545 24880859 PMC4062427

[52] Gabriel LR, Wu S, Kearney P, Bellvé KD, Standley C, Fogarty KE, et al Dopamine transporter endocytic trafficking in striatal dopaminergic neurons: differential dependence on dynamin and the actin cytoskeleton. J Neurosci. 2013; 33: 17836–17846. 10.1523/JNEUROSCI.3284-13.2013 24198373 PMC3818556

[53] Wheeler DS, Underhill SM, Stolz DB, Murdoch GH, Thiels E, Romero G, et al Amphetamine activates Rho GTPase signaling to mediate dopamine transporter internalization and acute behavioral effects of amphetamine. Proc Natl Acad Sci USA. 2015; 112: E7138–7147. 10.1073/pnas.1511670112 26553986 PMC4697400

[54] Penmatsa A, Wang KH, Gouaux E. X-ray structure of dopamine transporter elucidates antidepressant mechanism. Nature. 2013; 503:85–90. 10.1038/nature12533 24037379 PMC3904663

[55] Liu JJ, Hezghia A, Shaikh SR, Cenido JF, Stark RE, Mann JJ. Regulation of monoamine transporters and receptors by lipid microdomains: implications for depression. Neuropsychopharmacology. 2018; 43: 2165–2179. 10.1038/s41386-018-0133-6 30022062 PMC6135777

[56] Chaudhuri A, Bhattacharya B, Gowrishankar K, Mayor S, Rao M. Spatiotemproal regulation of chemical reactions by active cytoskeletal remodeling. PNAS. 2011;108: 14825–14830. 10.1073/pnas.1100007108 21873247 PMC3169122

[57] Rosholm KR, Leijinse N, Mantsiou A, Tkach V, Pedersen SL, Wirth VF, et al Membrane curvature regulates ligand-specific membrane sorting of GPCRs in living cells. Nat Chem Biol. 2017; 13:724–729. 10.1038/nchembio.2372 28481347

