# Supplementary figures and images for: Quantum dots reveal heterogeneous membrane diffusivity and dynamic surface density polarization of dopamine transporter
Source: PLoS One. 2019 Nov 21;14(11):e0225339. doi: 10.1371/journal.pone.0225339 (PMC6872175; doi:10.1371/journal.pone.0225339)

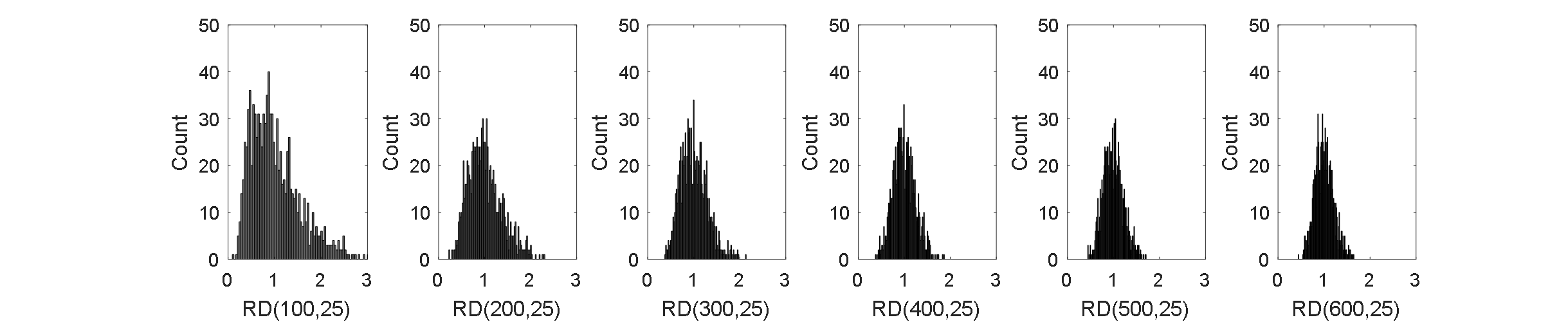

Supplement: S1 Fig — Histograms of trajectory number for deduced RD(N,25) for 1,000 simulated Brownian (free/nonanomalous) trajectories with varying track length N = 100, 200, 300, 400, and 600. (TIF) [file pone.0225339.s004.tif]

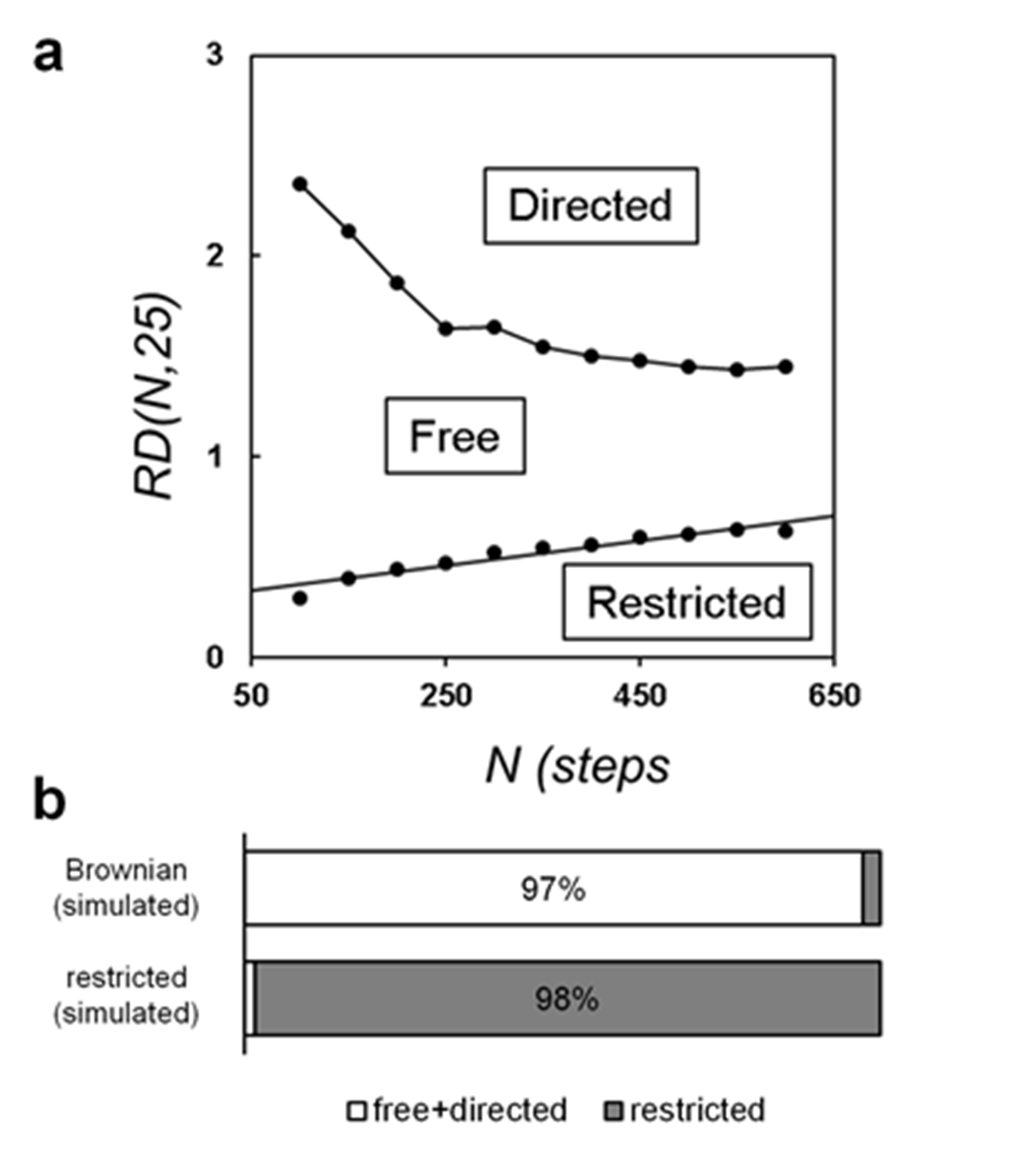

Supplement: S2 Fig — (a) Solid circles indicate 2.5th and 97.5th percentiles. (b) Computed fractions of simulated trajectories (each containing 1,000 tracks of varying length, i.e. 50–600 frames) classified as undergoing free diffusion (white) versus restricted diffusion (gray). (TIF) [file pone.0225339.s005.tif]

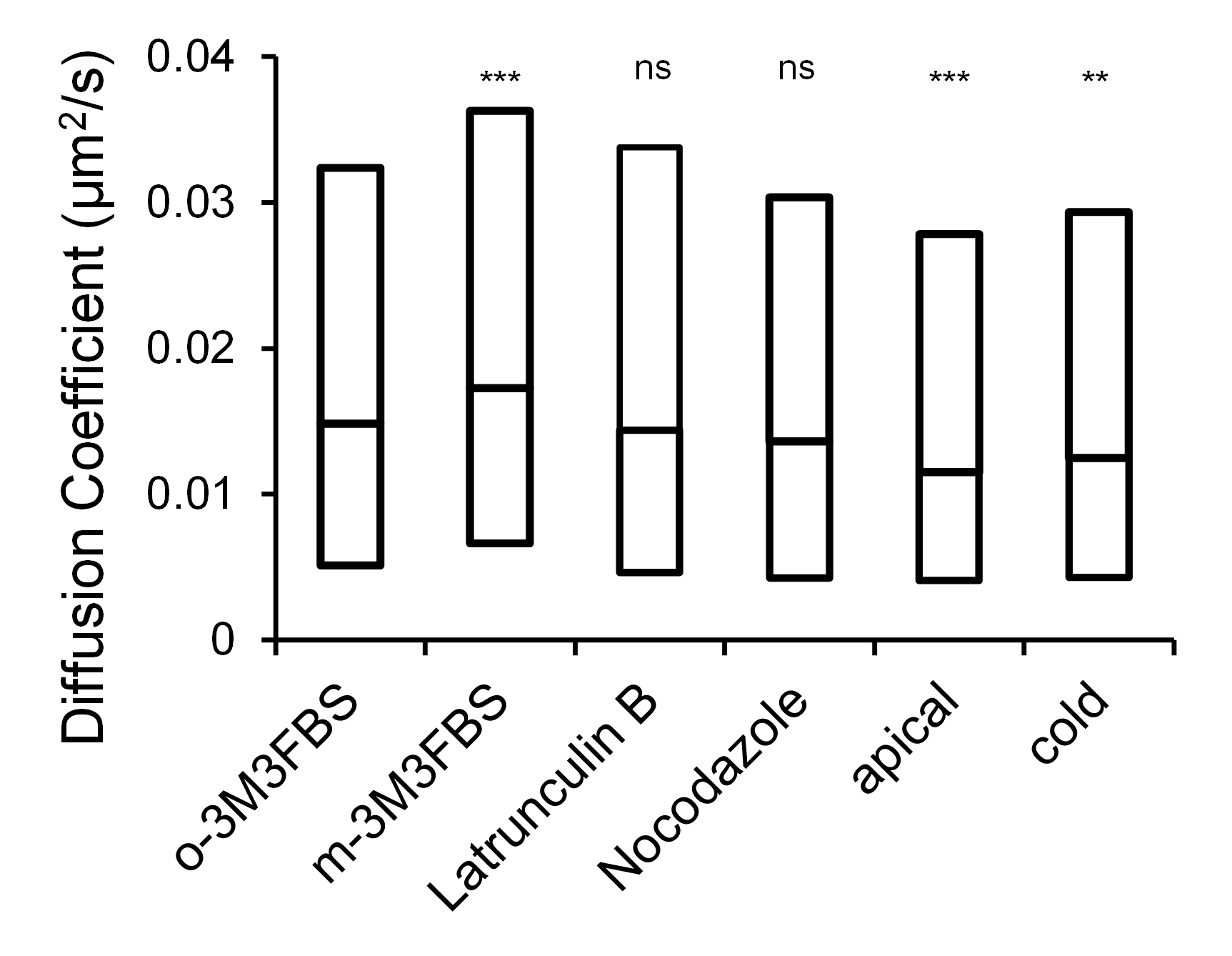

Supplement: S3 Fig — Trajectories of Qdot-tagged RFP-DAT-WT were recorded a spinning disk confocal microscope at 10 Hz for 1 minute (a total of 2767 (o-3MF3BS), 3932 (m-3M3FBS), 3311 (Latrunculin B), 3173 (Nocodazole), 924 (apical membrane), and 3867 (cold imaging buffer) tracks ≥50 frames were analyzed from three independent experiments for each conditions. Differences between Dmle distributions for each condition versus the control group were tested for statistical significance using the Kolmogorov-Smirnov test (*** denotes p<0.001, ** denotes p<0.01). (TIF) [file pone.0225339.s006.tif]

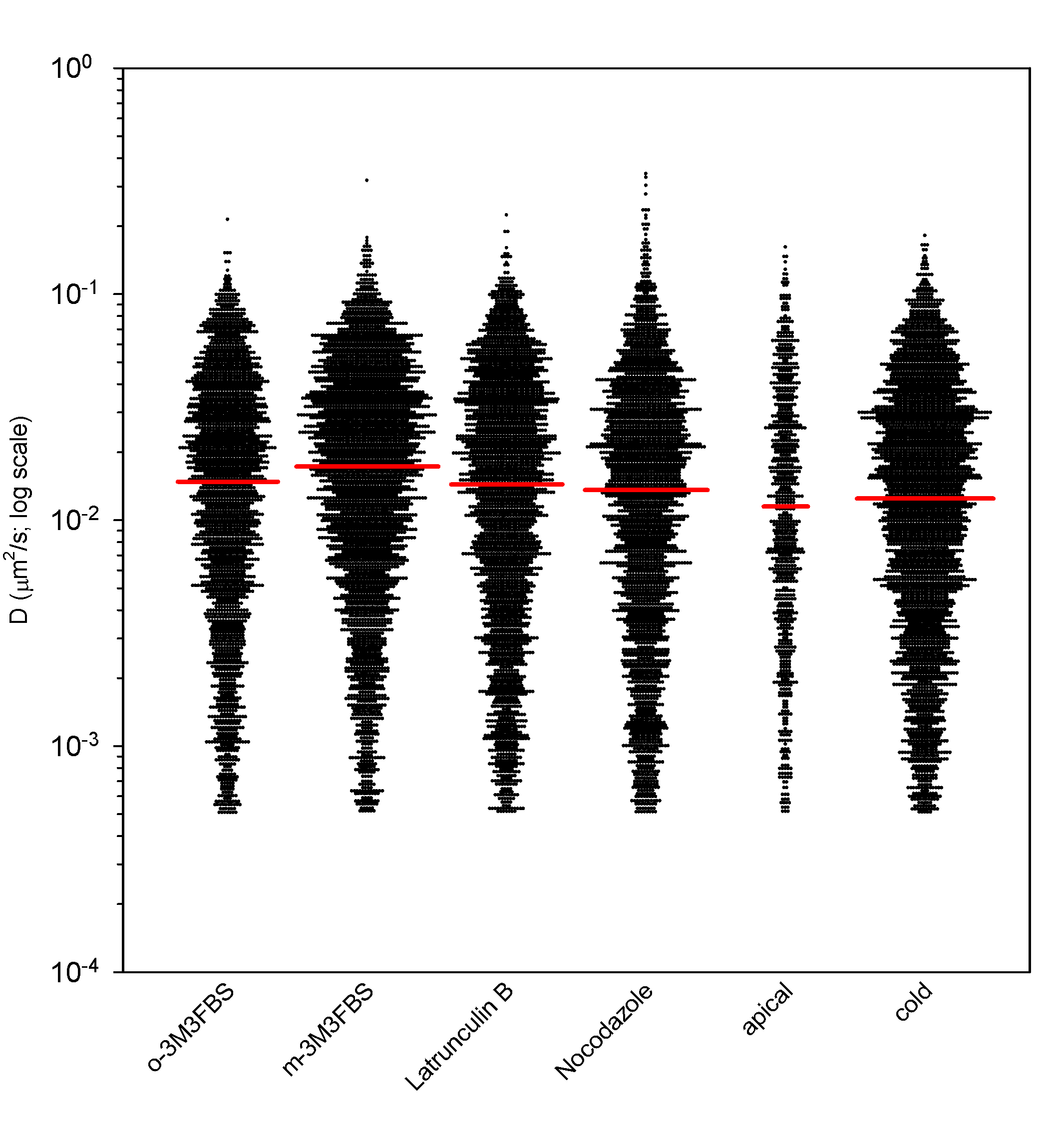

Supplement: S4 Fig — The median is represented by the black line. (TIF) [file pone.0225339.s007.tif]

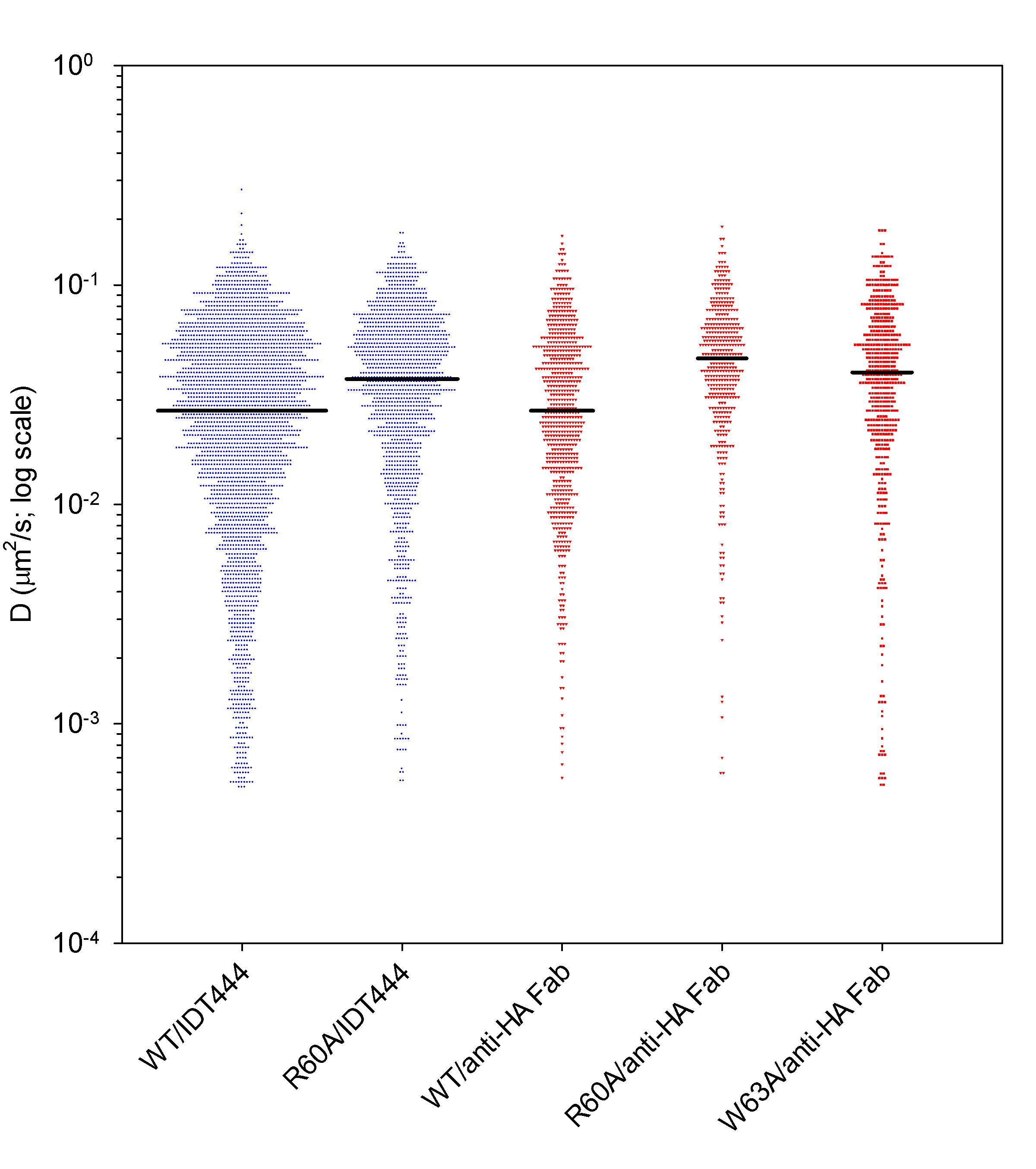

Supplement: S5 Fig — Median is represented by the black line. (TIF) [file pone.0225339.s008.tif]
